# Genomic characterization and histologic analysis of uterine leiomyosarcoma arising from leiomyoma with bizarre nuclei

**DOI:** 10.1002/path.6379

**Published:** 2024-12-18

**Authors:** Christopher Felicelli, Xinyan Lu, Zachary Coty‐Fattal, Yue Feng, Ping Yin, Matthew John Schipma, Julie J Kim, Lawrence J Jennings, Serdar E Bulun, Jian‐Jun Wei

**Affiliations:** ^1^ Department of Pathology Northwestern University Feinberg School of Medicine Chicago IL USA; ^2^ Department of Obstetrics and Gynecology Northwestern University Feinberg School of Medicine Chicago IL USA; ^3^ Lurie Cancer Center Northwestern University, Feinberg School of Medicine Chicago IL USA

**Keywords:** copy number alteration, digital image analysis, immunohistochemistry, leiomyoma with bizarre nuclei, oncogene sequencing, spatial transcriptome, tumor progression, uterine leiomyosarcoma

## Abstract

Leiomyoma with bizarre nuclei (LM‐BN) is a rare variant of leiomyoma with a benign clinical course. In contrast, leiomyosarcoma (LMS) is a high‐grade, malignant neoplasm characterized by high recurrence rates and poor survival. While LM‐BN and LMS show distinct morphologies, they share similar immunoprofiles and molecular alterations, with both considered ‘genomically unstable’. Rare cases of LM‐BN associated with LMS have been reported; however, the histogenesis and molecular relationship between these two tumors remains unclear. In this study, we assessed 11 cases of LMS arising in conjunction with LM‐BN confirmed by histology and immunohistochemistry (IHC), further analyzed by clinical, histologic, and molecular characteristics of these distinct components. LM‐BN and LMS had similar p16 and p53 IHC patterns, but LMS had a higher Ki‐67 index and lower estrogen and progesterone eceptor expression. Digital image analysis based on cytologic features revealed spatial relationships between LMS and LM‐BN. Genomic copy number alterations (CNAs) demonstrated the same clonal origin of LMS arising from existing LM‐BN through conserved CNAs. LMS harbored highly complex CNAs and more frequent losses of the *TP53*, *RB1*, and *PTEN* genomic regions than LM‐BN (*p* = 0.0031), with *CDKN2A/B* deletion identified in LMS only. Mutational profiling revealed many shared oncogenic alterations in both LM‐BN and LMS; however, additional mutations were present within LMS, indicative of tumor progression through progressive genomic instability. Analysis of spatial transcriptomes defined uniquely expressed gene signatures that matched the geographic distribution of LM‐BN, LMS, and other cell types. Our findings indicate for the first time that a subset of LMS arises from an existing LM‐BN, and highly complex genomic alterations could be potential high risks associated with disease progression in LM‐BN. © 2024 The Author(s). *The Journal of Pathology* published by John Wiley & Sons Ltd on behalf of The Pathological Society of Great Britain and Ireland.

## Introduction

Uterine leiomyoma with bizarre nuclei (LM‐BN) is a rare variant of leiomyoma, and histologic evaluation remains the key method in the diagnosis of LM‐BN [1], and no reliable biomarkers can be used to clearly separate it from uterine leiomyosarcoma (LMS) [2, 3]. LM‐BN harbors recurrent genomic events that are commonly seen in LMS [4, 5]. LM‐BN is diagnosed at a mean age of 42.5 years, 10–15 years younger than patients with LMS [5]. LM‐BN is a benign variant of leiomyoma with occasional local recurrence, without disease‐related deaths [6, 7, 8]. However, LM‐BN with diffuse nuclear atypia and five to nine mitoses/10 high power fields (HPFs) poses a diagnostic concern, treated as a smooth muscle tumor of uncertain malignant potential (STUMP) [9, 10].

Uterine LMS is a high‐grade malignant neoplasm and histologically heterogeneous with three subtypes: epithelioid, myxoid, and spindle. While rare reports have detailed chemotherapeutic agents or radiation contributing to LMS development, the tumorigenesis of LMS has long been debated [11, 12]. Due to limited histological or molecular characterizations of LMS, the development of uterine LMS is considered a *de novo* event [13]. Few studies have suggested the presence of possible precursor lesions, including ‘myometrial dysplasia’, symplastic, or leiomyoma‐like areas within LMS [14, 15].

Studies have shown that both uterine LM‐BN and LMS are genomically unstable with DNA copy number alterations (CNAs) in common tumor suppressor genes including *TP53*, *CDKN2A/B*, *PTEN*, *RB1*, or genes involved in *AKT* pathways [16, 17, 18, 19]. While these shared molecular aberrations have been well characterized, none can be utilized in isolation to aid in diagnostic purposes or pinpoint etiologies or predict tumor progression, and therefore the identification of tumor origin of these two entities remains largely unknown [20]. Many LMS cases can show mixed areas of benign appearing symplastic or leiomyoma‐like areas, with cases showing no clear‐cut boundary between benign and malignant components [15].

In this study, we aimed to examine the relationship and tumorigenesis of uterine LMS in relation to an adjacent LM‐BN counterpart and to identify similar and different molecular changes. We performed histologic, immunohistochemical, digital, and molecular profiling of both LMS and LM‐BN components from the same patients. We demonstrated that these cases possessed two distinct tumor components with unique histology, regional distributions, shared immunohistochemistry (IHC), and overlapping genomic alterations, particularly DNA CNAs. A spatial transcriptome analysis revealed cell‐specific and distinct expression profiles between LM‐BN and LMS regions, highlighting that these are distinct, rather than admixed, phenotypes. Both LM‐BN and LMS harbored many oncogenic mutations, with LMS gaining more mutations and CNAs than LM‐BN. These results suggest that significantly increased genomic instabilities in LM‐BN are highly associated with adjacent LMS.

## Materials and methods

### Case review (clinical, gross, and histologic features)

The study received approval by the Institutional Review Board. Cases were selected by retrospectively reviewing our LMS file, and cases with two distinct components of LM‐BN and LMS were included. Clinical information was summarized in supplementary material, Table S1. Cases with ill‐defined ‘leiomyoma area’ within LMS were excluded in this study. All available slides for each case (range: three to 69 slides, mean slides 30.1/case) were reviewed by gynecologic pathologists. Histologic assessment included tumor type, cellularity, nuclear atypia, mitotic count, necrosis, infiltration, and stage. Unique areas of distinct LMS and LM‐BN regions were determined based on WHO 2020 [10] and Blaustein's Pathology of the Female Genital Tract [21]. The histologic criteria for LM‐BN were well‐ demarcated tumor regions with high‐ or low‐density nuclear atypia in a diffuse or patchy pattern with a low mitotic index (all except one were <5/10 HPFs) and no tumor necrosis. One case (case 2) with six mitoses/10 HPFs at time of diagnosis was classified as LM‐BN, and now STUMP per new WHO criteria. The histologic criteria for spindle cell LMS included at least two of the following: hypercellular tumor regions with marked cytologic atypia, high mitotic index (≥10 mitoses/10 HPF), or tumor coagulative necrosis. HPF (high power field): 40× objective, field diameter = 0.55 mm, 10 HPFs = 2.4 mm^2^. Demographic and clinical characteristics recorded included age, history, radiology, surgical procedure, and outcomes.

### Immunohistochemical analysis

Sections of selected blocks, including both LMS and LM‐BN components, were subjected to IHC analysis with a panel of IHC stains: p16 (E6H4 clone), p53 (Bp53.11 clone), ER (SP1 clone), PR (1E2 clone), and Ki‐67 (30–9 clone). Detailed antibody information for this panel and other relevant markers was summarized in supplementary material, Table S2A. IHC was performed using a Ventana Nexus automated system, as described previously [22, 23]. Intensity was scored as absent (0), weak (1+), moderate (2+), or strong (3+), and the percentage of positive tumor cells was scored from 0% to 100%. p53 interpretation was either diffuse mutant (>75% of tumor), null mutant, or wild type (WT). p16 expression was assessed as patchy or diffuse (cytoplasmic and nuclear strong staining in >80% of tumor).

### Digital AI image analysis of growth pattern and relationship

Select slides were scanned using a Leica Aperio GT450 slide scanner. Whole‐slide images were saved as SVS files. Whole‐slide images of H&E slides were analyzed utilizing QuPath open‐source software [24]. Tumor cells were identified using the cell detection analysis algorithm. Five regions for each of the LMS, LM‐BN, and myometrium/leiomyoma (MM/LM) components were annotated at 40× magnification, corresponding to ~1% of total tumor volume/slide. Object classifier analysis was performed on the slides to generate spatial mapping of distinct regions (semantic segmentation). The regions of interest were classified as follows: red, LMS; yellow, LM‐BN; and green, MM/LM. Regions 1 × 1 mm square were analyzed for each component in quintuplet to assess nuclear and cytoplasmic features.

### Genomic DNA copy number profiling

Tumor sections from LM‐BN and LMS were marked by pathologists and dissected by technicians under the supervision of molecular pathologists. DNA from each region was extracted independently. DNA was analyzed using Affymetrix OncoScan CNA arrays (Thermo Fisher Scientific, Santa Clara, CA, USA). The data were analyzed with normal pool genomic DNA reference using Affymetrix Chromosome Analysis Suite (ChAS) software and reviewed by cytogeneticists for CNAs, including copy number gains or losses, and copy neutral loss of heterozygosity (CN‐LOH) (for details see Supplementary materials and methods).

### Spatial transcriptomic RNA sequencing and data analysis

Cases with the best distributions of both LMS and LM‐BN back to back within 6 × 6 mm regions were selected for spatial transcriptomic RNA sequencing (RNA‐seq). RNA quality was examined using an Agilent Bioanalyzer RNA pico‐chip (Santa Clara, CA, USA) to confirm that DV200 > 30%. Tissue morphology was examined on H&E‐stained slides to confirm the presence of both LMS and LM‐BN components. Deparaffinization, H&E staining and imaging, and decrosslinking of tissue sections were performed following 10× Genomics protocol (CG000520 Rev B) specific to the Visium CytAssist spatial gene expression (Pleasanton, CA, USA) for FFPE kit. Then the slides subjected to human probe (version 2) hybridization and ligation using 10× Genomics Visium CytAssist spatial gene expression FFPE, human transcriptome, 6.5 mm kit (10× Genomics, PN‐1000520). The probes were released from the tissue slide and transferred to a barcoded Visium slide using a Visium CytAssist instrument followed by probe extension. Sequencing libraries were prepared following the manufacturer's protocol. Multiplexed libraries were pooled and sequenced using a Novaseq6000 at the Northwestern University NUseq core facility. Method details are summarized in Supplementary materials and methods.

Raw sequencing data, in base call format (.bcl), were demultiplexed using Space Ranger (version 2.0.0) from 10× Genomics, converting the raw data into FASTQ format. The images were processed and manually aligned using the Loupe Browser (version 5.1.0). Space Ranger was also used for the alignment of the FASTQ files to the human probe set version 2 and to count the number of reads from each cell that aligned to each probe. The resulting matrix files, which summarize the alignment results, were imported into Seurat (Satija Lab, New York, NY, USA) for further analysis. In Seurat, each individual sample was preprocessed, normalized, and scaled. All samples were then combined into a single dataset using the ‘IntegrateData’ function in Seurat, adding metadata with the original sample information. All UMAPs, violin plots, spatial feature expression plots, and heatmaps were generated using Seurat tools. Cell types were automatically assigned using the R package scType.

### Next‐generation sequencing (NGS)

For exon sequencing, DNA libraries were prepared and performed using the BGISEQ‐500 platform (BGI, Cambridge, MA, USA). Rolling circle amplification (RCA) was performed, qualified captured libraries were loaded onto the BGISEQ‐500 platform, and high‐throughput sequencing was performed. Raw data were filtered by removing error sequences. The clean sequence data from each sample were mapped to the human reference genome (GRCh37/HG19) using Burrows–Wheeler Aligner (BWA) software (http://bio-bwa.sourceforge.net) [25] with a data analysis quality control (QC) system.

The 700 customized oncogene panel sequencing was performed at the Northwestern Diagnostic Molecular Biology Laboratory. The DNA library was hybridized to bridge probes targeting 700 genes. Bridge probes were captured with an anchor probe labeled with biotin and magnetic streptavidin‐coated beads. The library was purified, quantified, and normalized into a sequencing pool and sequenced using a NovaSeq® 6000 and a S1 flowcell (Illumina, San Diego, CA, USA). Sequence data were aligned using BWA1 (version 0.7.17) to human genome version hg38 (Human genome build 38). Variants were called using Vardict2 (version 1.8.3) and Pindel3 (version 0.2.5b). Variants were annotated using wAnnovar4 [25]. Additional details are provided in Supplementary materials and methods.

The data were analyzed as follows: reads from cleaned sequence data were mapped to the hg19 (UCSC) and DNA sequences from normal myometrium using BWA (version 0.7.5) with parameters ‘mem–t 8 –P –M’. Generated files were sorted, and PCR duplicates were removed using Picard (version 1.105) (http://broadinstitute.github.io/picard). Subsequently, the BAM files were indexed using samtools (version 0.1.19). The WGS had a quality score of 20 (Q20), and 95–96% of reads were successfully aligned (supplementary material, Figure S2).

### Statistical analysis

Statistical analysis was performed in GraphPad Prism (GraphPad Software, San Diego, CA, USA) utilizing unpaired *t*‐tests and one‐way ANOVA. *P* values less than 0.05 were considered statistically significant, and *p* values were defined as follows: *p < 0.05, **p < 0.01, ***p < 0.001, and ****p < 0.0001.

## Results

### Clinical and gross findings

Ten spindle cell LMS with adjacent LM‐BN and one LMS with adjacent STUMP were included in this study. The median age at the time of diagnosis was 47 (34–55). The median clinical follow‐up was 33.5 months. The mean tumor size was 8.9 ± 2.1 cm, and three cases (3/11, 27.3%) presented with metastatic disease. Six cases were at FIGO stage I, two stage II, one stage III, and two stage IV. Two patients died of disease (18.2%), and among nine patients alive, one had disease recurrence while the remainder are disease free with median follow‐up of 27 months (5–107 months). Six (54.5%) cases had separate leiomyoma(s). The demographic and clinical characteristics are summarized in Table 1 and supplementary material, Table S1.

**Table 1:** Clinical characteristics of 11 LMS in conjunction with LM‐BN.

Cases	Age (years)	Race	Tumor size (cm)	Metastasis or extension	Stage	LMS‐specific survival	Follow‐up (months)
Case 1	43	Asian	27	Pelvic	II	Alive	44
Case 2	42	Black	6	Lungs, brain	IV	Dead	107
Case 3	48	White	7.8	No	III	Alive	33
Case 4	41	White	3.7	Pelvic	IIB	Alive	27
Case 5	54	White	10.5	No	IB	Dead	26
Case 6	55	White	14.9	No	IB	Alive	36
Case 7	50	White	4.5	No	IA	Alive	34
Case 8	55	White	7.5	No	IB	Alive	25
Case 9	34	Black	3.5	No	IB	Alive	12
Case 10	48	Black	3.7	No	IA	Alive	20
Case 11	43	Asian	9	Lungs, brain	IV	Alive	5
Median	48		8.9				27

### Microscopic and histologic findings

**Figure 1:**
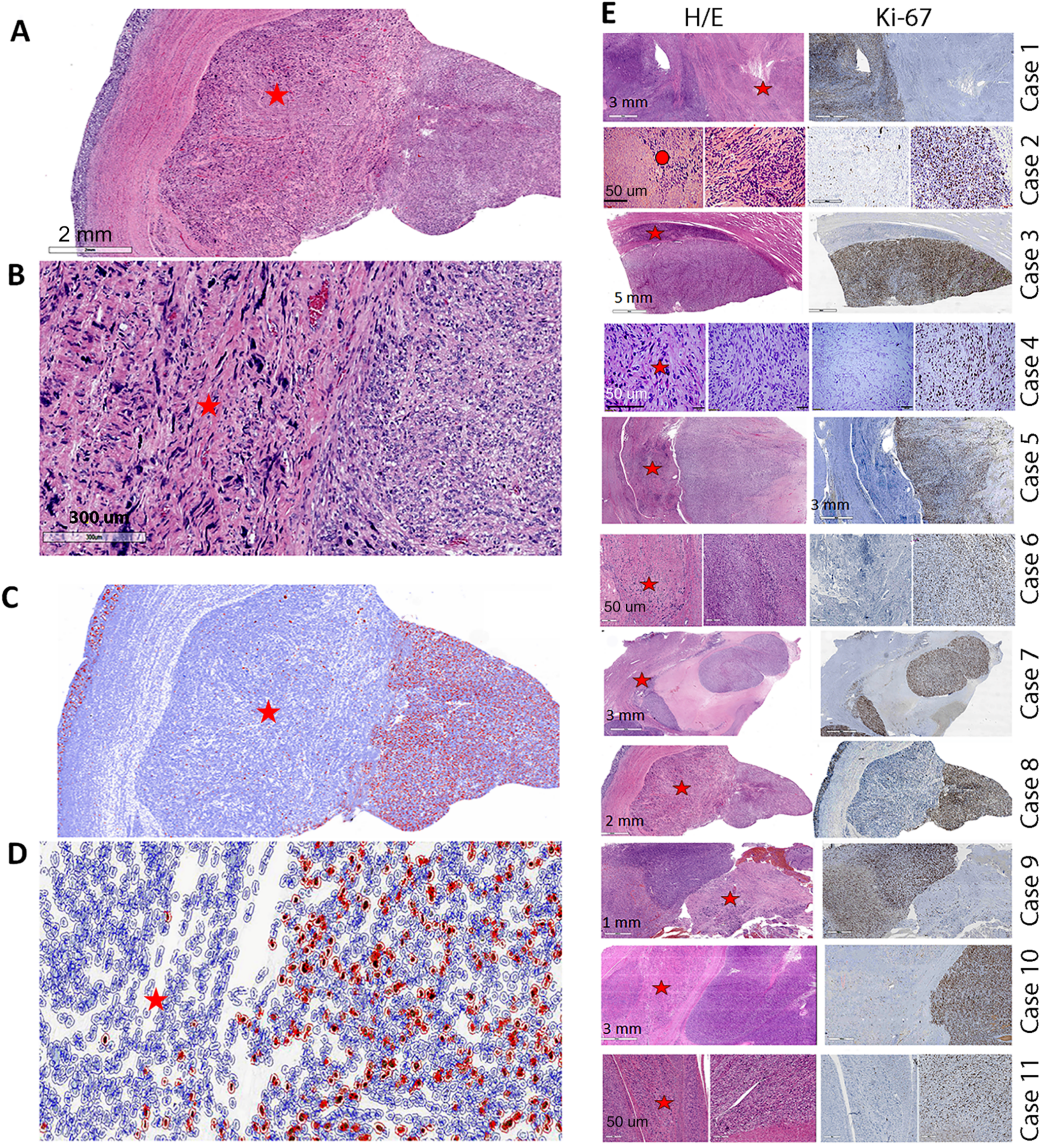
Photomicrographs illustrating 11 cases of LMS with adjacent LM‐BN. (A, B) Representative H&E section of case 8 demonstrating spatial distribution of hypercellular LMS (right) and LM‐BN (left) components in scanning power (A) and close view (B). (C, D) Digital images of case 8 captured by QuPath (see Materials and methods) highlighting tumor cells with different Ki‐67 index in LM‐BN and LMS components in scanning power (C) and close view (D). (E) H&E‐stained (left) and Ki‐67‐stained (right) sections of 11 cases of LMS with LM‐BN. Red stars mark LM‐BN regions and red circles mark STUMP. Scale bars labeled in millimeters or micrometers.

Microscopic analysis demonstrated the presence of distinct, well‐demarcated LMS and LM‐BN regions in all cases (Figure 1). Available imaging and gross pathology are illustrated in supplementary material, Figure S1. The spatial relationships of distinct regions of LM‐BN and LMS were most easily observed through side‐by‐side comparison of H&E‐ and Ki‐67‐stained slides, showing the unique distributions of LM‐BN and LMS regions (Figure 1A–D). The LMS regions showed hypercellular tumor cells with atypia, frequent mitoses, necrosis, and higher Ki‐67 index in comparison to LM‐BN regions. LM‐BN was characterized by symplastic‐like nuclear atypia, few mitoses, and absence of necrosis. All cases demonstrated the distinct spatial distribution of LM‐BN and LMS components, highlighted by H&E‐ and Ki‐67‐stained slides (Figure 1E). A closer view of histologic features in all 11 cases were summarized in supplementary material, Figure S2. The average tumor volume of LM‐BN components was 23% and 77% for LMS components. The density and distribution of nuclear atypia varied within LM‐BN regions, and mean mitotic counts were lower in LM‐BN (mean 1.7 ± 0.52) than in LMS (24.3 ± 2.78) (*p* <0.0001). Tumor necrosis was identified solely in the LMS components. The specific histological findings of each component are summarized in Table 2.

**Table 2:** Histologic features of LMS and LM‐BN or STUMP components.

Cases	LM‐BN				LMS			
	Density of nuclear atypia	Mitosis (/10 HPF)	Necrosis*	Percentage LMBN	Density of nuclear atypia	Mitosis	Necrosis*	Percentage LMS
						(/10HPF)		
Case 1	Intermediate	2	−	10	High	24	++	90
Case 2	High	6**	−	−	High	15	++	−
Case 3	Intermediate	1	−	5	High	12	+	95
Case 4	Low	0	−	20	High	20	+	80
Case 5	Intermediate	2	−	30	High	40	++	70
Case 6	Low	1	−	20	Low	21	+	80
Case 7	High	1	−	10	High	21	+	90
Case 8	High	1	−	15	High	30	+	85
Case 9	Low	1	−	−	High	36	+	−
Case 10	Low	2	−	20	Intermediate	24	+	80
Case 11	Intermediate	2	−	30	High	30	+	70
Mean		1.7 ± 0.52	0	16%		24.3 ± 2.78	100%	84%

*Tumor necrosis: −, negative; +, focal; ++, extensive: **, STUMP.

### Immunohistochemistry findings

**Figure 2:**
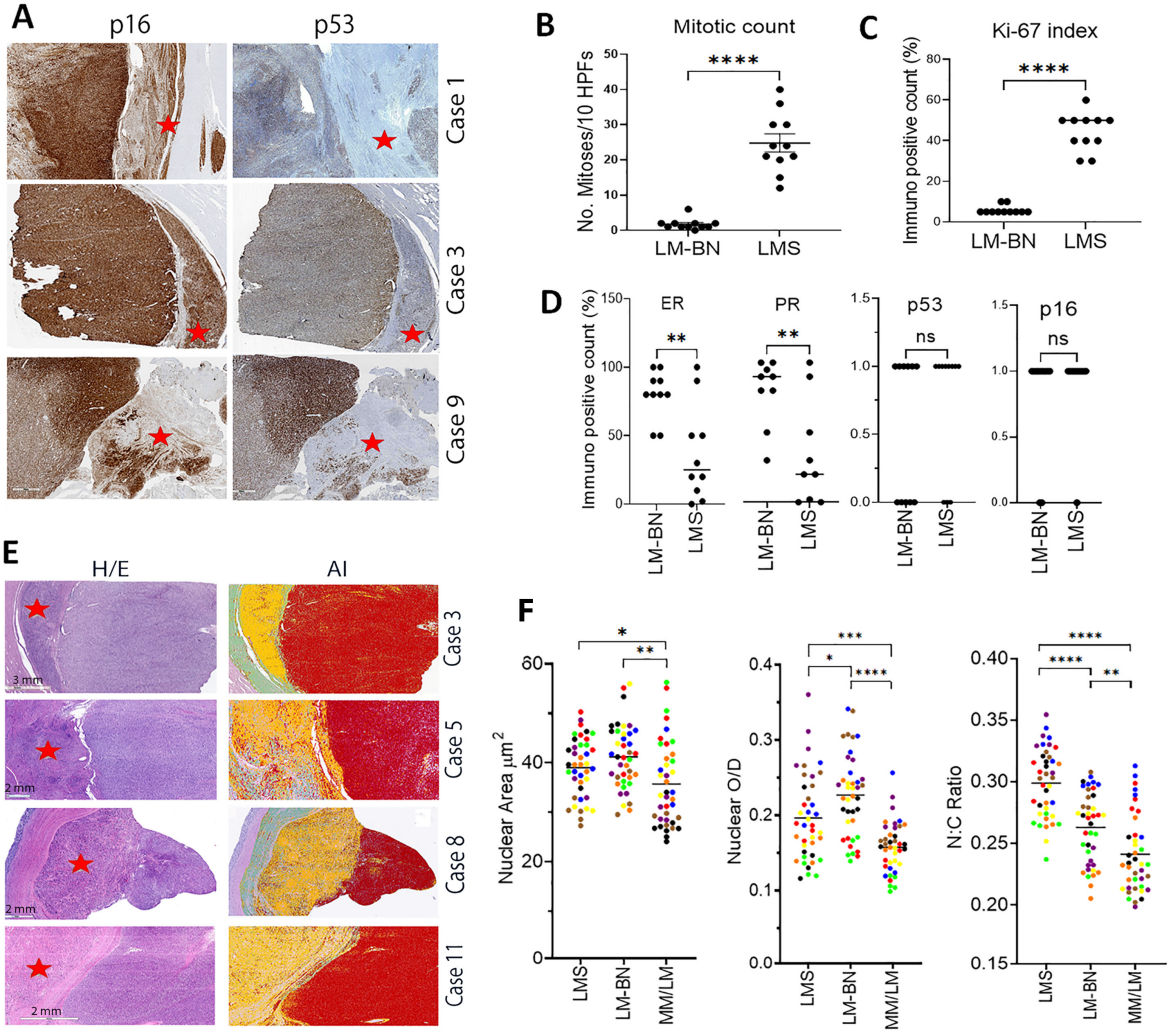
IHC and digital image analysis of LMS and LM‐BN components. (A) Representative sections showing immunostaining patterns for p16 and p53 in LMS (right) and LM‐BN (red star). (B, C) Dot plots illustrating differing mitotic index (B) and Ki‐67 index (C) between LM‐BN and LMS in all cases. (D) Dot plots summarizing immunoreactivity for percentage of ER and PR, for diffuse versus patchy stain for p16 and p53 in LMS and LM‐BN components. (E) H&E and digital image analysis by QuPath illustrate examples of spatial distribution of LMS (red), LM‐BN (yellow), and LM/myometrium (light green) based on nuclear and cytoplasmic features. (F) Dot plot analysis of nuclear area, chromatin density, and nuclear/cytoplasmic ratio in LMS, LM‐BN, and LM/myometrial components. Each dot represents data obtained in a 1‐mm^2^ target region, with differing colors representing different cases. *p <0.05; **p <0.01; ***p < 0.001; ****p < 0.0001; ns = no statistical significance.

All tumors underwent routine IHC to confirm the LMS diagnosis utilizing smooth muscle markers (SMA, desmin, or H‐caldesmon) and melanoma markers (HMB45, Melan A) to exclude PEComa. IHC for ER, PR, p16, and p53 performed in all available cases demonstrated similar expression patterns to previously published data for sporadic LMS and LM‐BN (Figure 2A–D and supplementary material, Table S2B) [8, 26, 27]. Mean Ki‐67 expression was significantly higher in LMS regions (44.5%) than LM‐BN regions (5.9%) (*p* < 0.001, Figure 2C). ER and PR expression was higher in LM‐BN (medium 80% and 79%) than LMS (37% and 35%) (*p* = 0.0029 and *p* = 0.0086, Figure 2D). Diffuse immunoreactivities for p16 and p53 were slightly higher in LMS (90.9%, 72.7%) than LM‐BN (81.8%, 54.5%) without statistical significance (Figure 2D). FH expression was present in all tumors.

### Digital image and AI analysis

To further solidify the spatial relationships between LMS and LM‐BN, QuPath cell detection and image classifier algorithms were applied to present whole‐slide images with regional annotation assistance. The cell classifier software defined cellular parameters and confirmed the presence of the spatial distribution of different histologic patterns. Based on the cytologic features, AI defined the distinct distribution patterns of LMS (red), LM‐BN (yellow), and LM/myometrium (green), and a color‐coded map was prepared from each case (Figure 2E). Small overlaps were identified, likely secondary to mild morphologic overlap that exists in cellular details. Cell detection analysis revealed subtle nuclear feature differences between LMS and LM‐BN (Figure 2F and supplementary material, Table S3). There were statistically significant differences in nuclear area between LMS and myometrium (*p* = 0.0486) and between LM‐BN and myometrium (*p* = 0.0017), with no significance between LMS and LM‐BN (*p* = 0.1237). LM‐BN had more substantial nuclear hyperchromasia compared to LMS (*p* = 0.0177) and myometrium (*p* < 0.0001), with LMS having more nuclear hyperchromasia compared to myometrium (*p* = 0.0004) Nuclear‐to‐cytoplasmic ratio was most increased in LMS compared to LM‐BN (*p* < 0.0001) and myometrium (*p* < 0.0001), as well as LM‐BN compared to myometrium (*p* = 0.0016).

### Assessment of DNA CNAs

**Figure 3:**
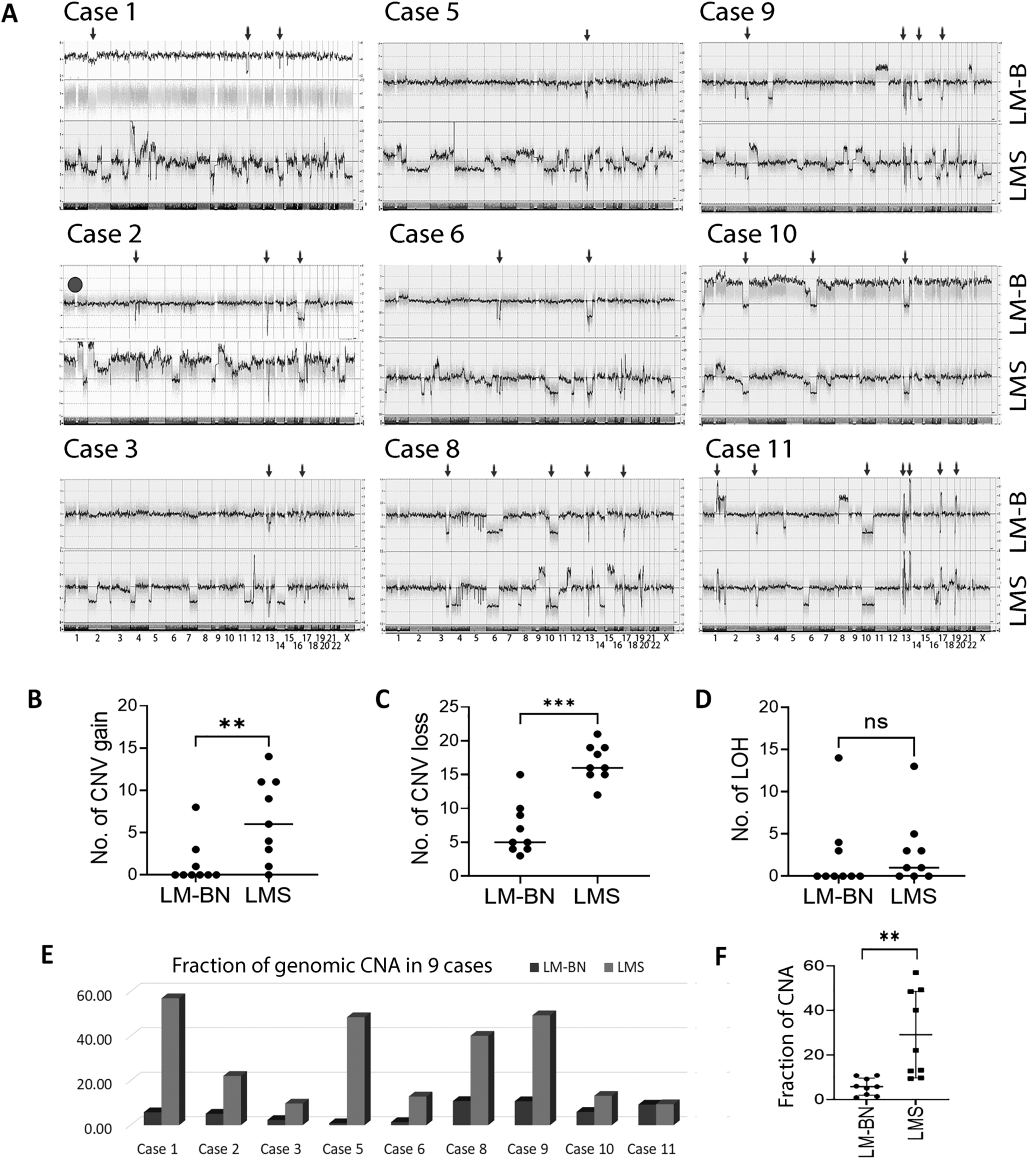
OSCHP plots by Affymetrix OncoScan for whole‐genome CNA. (A) Genomic copy number gain and loss for chromosomes 1 to 22 and X were obtained in nine cases, with LM‐BN regions presented above and LMS regions below. Arrows indicate CNA shared by LM‐BN and LMS, indicating clonality. (Case 1 LM‐BN was tetraploid. A circular dot in case 2 indicates STUMP component.) (B, C) Dot plots represented the number of detectable sites of (B) CNA gain and (C) loss in genomic regions from nine cases. (D) Dot plots for LOH in LM‐BN and LMS. (E, F) Fraction of genome alteration in LMS and LM‐BN regions (E) individually and (F) combined analysis of fraction of genome alteration in LMS and LM‐BN. *p <0.05; **p <0.01; ***p < 0.001; ns = no statistical significance.

Genome‐wide CNAs were analyzed by chromosomal microarray analysis in nine of 11 cases. DNA from annotated and macrodissected LM‐BN and LMS regions were prepared. CNAs and CN‐LOH plots between paired LMS and LM‐BN lesions were compared and analyzed (Table 3). The presence of shared CNA loci between LM‐BN and LMS was identified in all cases (Figure 3A), strongly suggesting the same origin of two tumor components. The mean site number of CNA is higher in LMS (27.11 ± 3.15) than in LM‐BN (12.22 ± 2.90) (*p* = 0.0031), (Figure 3B,C) and the mean fraction of genome alteration in LMS was 29.11% of genomic DNA (range 9.39–56.96%) in comparison to 5.79% (range 0.87–10.82%) in LM‐BN, indicating a higher level of DNA instability in LMS (Figure 3E,F, *p* = 0.0028). There was no difference in CN‐LOH (Figure 3D). The sites of total CNAs were 332, including 71 copy number gains, 214 copy number losses, and 47 CN‐LOH. The site and fraction of CNA was summarized in Figure 3F and supplementary material, Table S4.

**Table 3:** Relationship of CNAs in LMS and LM‐BN cases.

Case	LM‐BN		LMS		Shared changes	Major genes	Percentage CNA shared
	CNA	LOH	CNA	LOH	CNA/LOH		
1	Loss: 1q, 11q, 14q and 15q	Xq, 1p, 2p, 2q, 3q, 4p, 4q, 5q, 9p, 10q, 11p, 13q, 19q and 22q	Loss: 1p, 1q, 2q, 5p, 5q, 8q, 9p, 10q, 11q, 13q, 14q, 15q, 16q, 17p, 18p, 20p, 21p, 22q, and Xp Gain: 1q, 4p, 4q, 5p	Xq, 1p, 2q, 3q, 4p, 4q, 5q, 9p, 10q, 11p, 13q, 19q, and 22q	Loss: 1q, 11q, and 14q LOH: Xq, 1p, 3q, 4p, 4q, 5q, 9p, 10q, 11p, 13q, 19q and 22q	**CDKN2A/B**, PTEN, RB1, T53	41.7%
2	Loss: 4q, 5q, 7p, 7q, 13q, 14q, 16p, 16q, 17p, and 19q	Xp, 2p, 15q, and 20q	Loss: Xp, 2q, 4q, 5q, 6q, 7p, 7q, 9p, 10q, 13q, 14q, 16p, 17p, 19q, 21p, and 22q	2p, 15q, 20q	Loss: 4q, 5q, 7p, 7q, 13q, 14q, 16p, 16q, 17p, and 19q	TP53, PTEN, RB1	52.6%
3	Loss: 13q, 16q, 17p, and Xq		Loss: 1p, 2p, 2q, 4p, 5p, 7q, 11q, 12p, 13q, 14q, 16p, 16q, 17p, 17q, and Xq		Loss: 13q, 16q, 17p, and Xq		22.2%
			Gain: 8q, 12p, and Xp			TP53, RB1	
4	N/A	N/A	N/A	N/A	N/A	N/A	N/A
5	Loss: 11p, 11q, 12q, 13q, and 17p		Loss: 2p, 4q, 5, 6q, 9p, 10q, 11p, 11q, 12q, 12p, 13q, 14q, 15q, 16, 17p, 17q, 18, 19, 20p, and 22q Gain: X, 1p, 3p, 4p, 5p, 6p, 7, 8, 9p, 9q, 11p, 11q, 20q, and 21	1q, 6q, 11p/11q, 15q and 17q.	Loss: 11p, 11q, 12q, 13q, and 17p	TP53, RB1	12.8%
6	Loss: 6p, 6q, and 13q		Loss: Xp, Xq, 1p, 1q, 2q, 4q, 5q, 6p, 6q, 8q, 9q, 10p, 10q, 11q, 12p, 13q, 16p, 16q, 17p, 19q, and 20p Gain: 3p, 10, 16q, 17p, 17q, and 19q		Loss: 6p, 6q, and 13q	TP53, PTEN, RB1	11.1%
7	N/A	N/A	N/A	N/A	N/A	N/A	N/A
8	Loss: Xq, 3q, 4q, 5q, 6p, 6q, 10q, 13q, and 17p Gain: 19p		Loss: Xq, 3q, 4p, 4q, 5q, 6p, 6q, 8p, 10p, 10q, 13q, 14q, 16q, 17p, 19p, and 19q Gain: 2q, 9p, 9q, 11q, 13q, 15q, 17p, 19p, and 19q	6q	Loss: Xq, 3q, 4q, 5q, 6p, 6q 10q, 13q, and 17p Gain:19p	TP53, PTEN, RB1	49.6%
9	Loss: 2q, 4p, 6p, 7p, 13q, 14q, and 17p		Loss: Xp, Xq, 2q, 5q, 6p, 7q, 9p, 10q, 11p, 13q, 14q, 16p, 17q, 19p, and 19q	17p	Loss: 2q, 6p, 13q, and 14q	TP53, RB1	18.5%
	Gain: 11, 16p, and 21		Gain: 1q, 2q, 3p, 7q, 8q, 9q, 13q, 16p, 17q, 19p, and 19q		Gains: 16p		
10	Loss: 1p, 2p, 6p, 6q, and 13q		Loss: 1p, 2p, 6p, 6q, 7q, 9p, 9q, 11q, 13q, 16q, 17p, and 20q Gain: 1q		Loss: 1p, 2p, 6p, 6q, and 13q	RB1	38.5%
11	Loss: 1p, 2q, 3q, 4p, 6p, 7q, 8p, 8q, 10q, 13q, 14q, 16q, 17p, 18p, and 20p Gain: Xp, 2p, 13q, 16p, 17p, 18q, 20p, and 20q	Xq, 15q, and 17p	Loss: 1p, 2q, 3q, 4p, 4q, 6p, 7q, 8p, 8q, 10q, 13q, 14q, 16p, 17p, 18p, 19p, 20p, and 22q. Gain Xp, 2p, 4q, 7p, 8q, 16p, 17p, 18q, 19q, 20p, and 20q.	Xq, 15q, and 17p	Loss: 1p, 2q, 3q, 4p, 6p, 7q, 8p, 8q, 10q, 13q, 14q, 16q, 17p, 18p, and 20p Gain: Xp, 2p, 13q, 16p, 17p, 18q, 20p, and 20q	RB1, TP53	76.3%
	Chromothripsis: 8 and 13		Chromothripsis: 4, 8, and 13		LOH: Xq, 15q, and 17p		
	Amplifications: 17p and 18q		Amplifications: Xp, 17p, and 17q		Chromothripsis: 8 and 13		
					Amplifications: 17p		

The common CN losses in LM‐BN component were 13q (*RB1*, 88.9%), 17p (*TP53*, 66.7%), 6p (55.5%), and 14q (44.4%), and in LMS component were 13q (*RB1*, 100%), 10q (*PTEN*, 77.8%), 17p (*TP53*, 88.9%), 14q (77.8%), 2q (66.7%), 1p, 4q, 5q, 6q, 7, 9p, and 16q (55.5%), and the common CN gains in LMS were 1q, 2q, 8q, 9q, 13q, 17p, and 17q (33.3%). Of all CNAs observed, the average percentage of shared CNAs between LM‐BN and LMS regions was 29.4% (11.1–76.3%), suggesting LM‐BN and LMS lesions in examined cases had same tumoral origin.

The most common shared loci between LMS and LM‐BN were losses in 13q (88.9%), 17p (66.7%), 6p (55.5%), 14q (44.4%), 6q (33.3%), 10q (33.3%), and 16q (33.3%). CN losses accounted for the vast majority of CNAs, accounting for 68.1% of LM‐BN, 65.4% of LMS, and 66.1% of overall CNAs. Notably, these shared CN losses showed several specific genomic alterations in chromosomal loci encoding major genes in tumorigenesis, including *PTEN* (10q23.31, 11.1% in LN‐BN versus 44.4% in LMS), *RB1* (13q14.2) (77.8% in LN‐BN versus 100% in LMS), and *TP53* (17p13.1, 66.7% in LN‐BN versus 88.9% in LMS). CN loss of *CDKN2A/2B* (9p21.3) was only observed in LMS (11.1%) (Table 3). Additional CNAs only identified within LMS included alterations in 3p, 5p, 9q, 10p, 12p, 17q, and 21p.

### Spatial transcriptome analysis (RNA‐seq)

**Figure 4:**
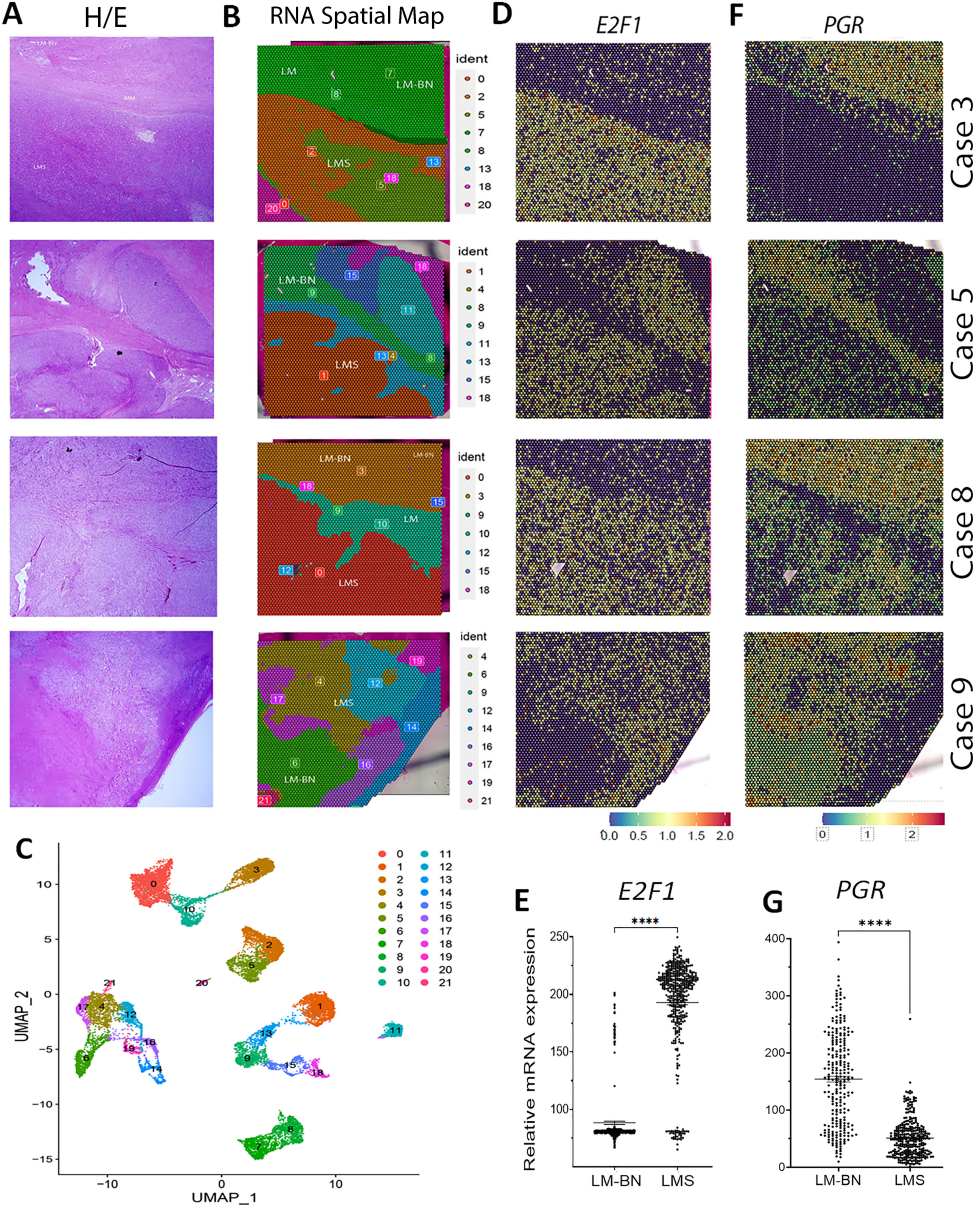
Spatial transcriptomic analysis of LMS and LM‐BN. (A, B) Side‐by‐side comparison of H&E‐stained tumor sections of (A) different tumor components with (B) spatial transcriptomic maps. The 0–21 RNA signatures of different colors are listed on right side. (C) UMAP cluster analysis represents distance and proximity of 0–21 RNA signatures, corresponding to differing gene expression signatures (see results for detail). (D–G) Spatial RNA expression maps of representative genes (D) *E2F1* and (F) *PGR* illustrated with color bars (below), indicating levels of gene expression by region. Dot plot analysis demonstrates relative RNA expression differences between LM‐BN and LMS regions of (E) *E2F1* and (G) *PGR*. Each dot represents a single transcriptomic dot captured by QuPath. ***p < 0.001.

Sections from four cases of LMS with adjacent LM‐BN that passed RNA quality testing were utilized for spatial transcriptome analysis (supplementary material, Figure S4). The depth of reads with spots in tissue is summarized in supplementary material, Table S5A. Spatial transcriptome analysis highlighted distinctly defined geographical patterns of transcription in LM‐BN, LMS, and MM/LM. Importantly, the spatial transcriptome map matched the tumor region and type with the corresponding histology (Figure 4A,B). Unsupervised analysis identified a total of 21 clusters across the four cases (Figure 4C), highlighting distinct regional variances based on RNA signatures. Two top ranking genes, *E2F1* (checkpoint regulator) and *PGR* (progesterone receptor), in all four cases were selected as examples for electronic in situ analysis that revealed distinct expression patterns between LMS and LM‐BN regions, demonstrating their unique geographical transcription differences (Figure 4D,F). Quantitative analysis showed significant differences of these two genes between LMS and LM‐BN regions (*p* < 0.001, Figure 4E,G).

**Figure 5:**
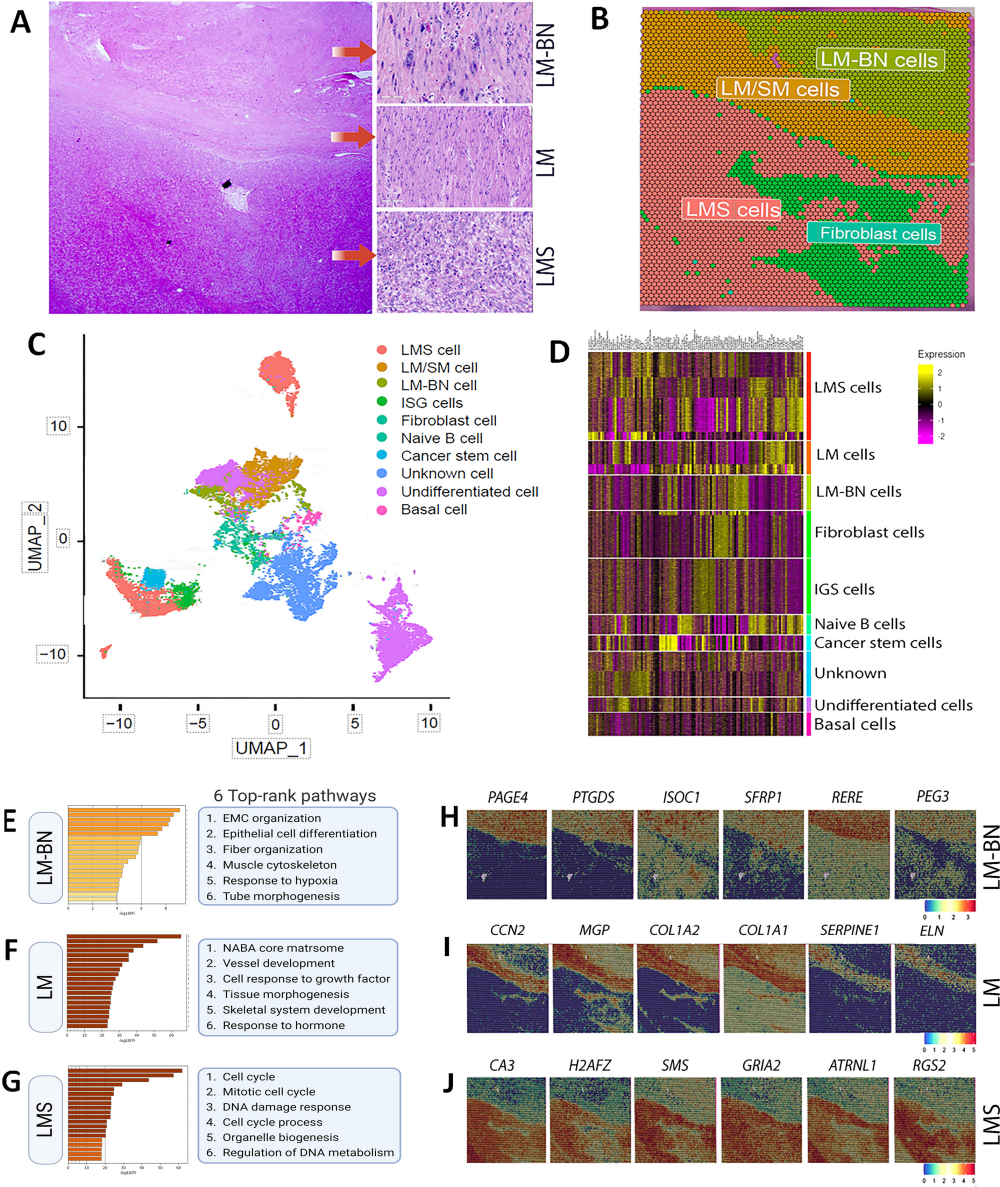
Spatial transcriptomic analysis of RNA signature in different cell types and major gene pathways in case 3. (A) H&E section of case 3 demonstrating LMS, LM, and LM‐BN components with close view in red arrows. (B) Spatial transcriptomic map recapitulating distribution of dominant cell types by expression signature. (C) UMAP cluster analysis illustrating graphic clusters of 10 cell types and abundance detected by cell‐specific RNA signatures. (D) Supervised gene cluster analysis and heatmap revealed specific gene expression patterns in each of 10 cell types, with color bars representing relative gene expression levels. (E–G) KEGG pathway analysis demonstrating top‐ranking pathways presented in (E) LM‐BN, (F) LM, and (G) LMS components. (H–J) Spatial gene expression maps demonstrating gene expression patterns for selected top‐ranking genes in (H) LM‐BN, (I) LM, and (J) LMS components, with color bars indicating expression level in bottom right corner.

To define the zonal difference of spatial transcriptome between LM‐BN and LMS, case 3 was subjected to heightened analysis of cell‐type‐specific transcription [28]. When the gene signatures were defined in 10 specific cell types, the regional distribution of the cell types was highly correlated with histologic cell types (Figure 5A,B). As expected, LMS cells showed more heterogeneity in cluster analysis (Figure 5C) and presented four gene signatures (Figure 5D). Of note, LM‐BN regions showed two gene signatures (Figure 5C,D). The LMS region contained fibroblast, stemness, undifferentiated, or unknown cell natures, suggesting a complex nature of tumor differentiation (Figure 5D). This might be highly relevant to the complex CNA in LMS (Figure 3A). Pathway analysis revealed different functional pathways between LMS. MM and LM‐BN (Figure 5E–G), and the six top‐ranking pathways in LMS correlated with aggressiveness and progression of tumor nature (Figure 5E).

To further analyze the gene expression, some top‐ranking genes from LM‐BN (Figure 5H), LM (Figure 5I), and LMS (Figure 5J) were selected for spatial geographic expression analysis, and distinct gene expression patterns were highly correlated with the histologically defined tumor natures. The global and spatial transcriptome demonstrated the different gene expression profiles among LM, LM‐BN, and LMS, indicative of the different stages of tumorigenesis.

### Mutational profiling on oncogene panel

**Figure 6:**
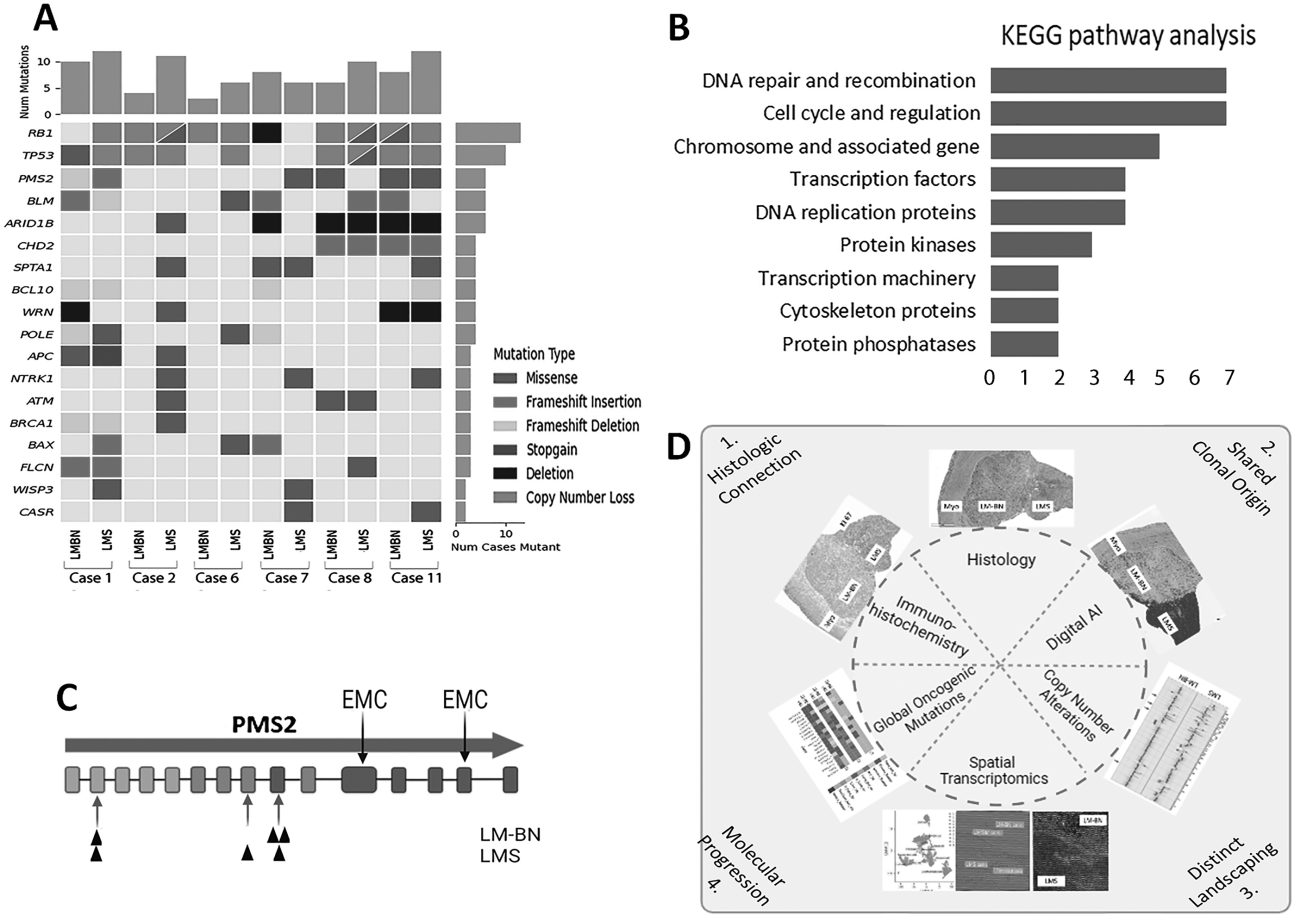
Landscape of oncogenic mutations in LMS with LM‐BN. (A) Co‐mutation plot diagram illustrating 18 top‐ranking oncogenes frequently mutated in six selected cases of LMS and LM‐BN components, revealing many identical mutations between LMS and LM‐BN (supplementary material, Table S6A). (B) Pathway analysis by KEGG revealing major oncogenic pathways regulated by these genes. (C) Detected mutation sites for PMS2 for LMS and LM‐BN components (black triangle below exon), with common mutation sites for endometrial cancer (EMC) marked above exons. (D) Graphical diagram summarizing cumulative multimodal morphologic and molecular analysis of LMS with LM‐BN (created with BioRender.com).

To evaluate the connection and tumor progression of LMS from existing LM‐BN, gene mutation analysis from 700 oncogene panels or exon sequencing was performed in six cases (supplementary material, Table S6B). Variant calling was subjected to analysis and referred to established variant libraries (ClinVar, OncoKB, ClinGen). The top 18 mutated genes were selected for further analysis. As illustrated in Figure 6A, this subset of oncogenes and tumor suppressors was frequently mutated in both LM‐BN and LMS, with significant overlap. While mutant genes were very different in each case, there were trends of shared oncogenic mutations between LM‐BN and LMS components, defined as identical nucleotide changes. Some genes were found to be mutated in either LM‐BN or LMS, but not both, suggesting subclonal mutation events (supplementary material, Table S6A). Of note, LMS accumulated more oncogenic mutations than LM‐BN. The high rate of *RB1* and *TP53* alterations, by either CNA or mutation, in both LM‐BN and LMS is consistent with previous studies [29, 30, 31]. Pathway analysis showed that the major oncogenic genes were involved in DNA damage response and cell cycle alteration (Figure 6B). Several oncogenes frequently mutated in endometrial and ovarian cancer (*ARID1B*, *ATM*, *BRCA1*, *CHD2*, *POLE*, and *PMS2*) were also present in LMS. *PMS2* was frequently mutated in four of six cases (Figure 6C). *NTRK1* missense mutations were found in three of six LMS components; however, they were not identified within LM‐BN components. The patterns of mutations in these two components suggest (1) there is a wide spectrum of gene mutations in both LM‐BN and LMS; (2) most gene mutations are likely secondary events; and (3) many shared mutations between LM‐BN and LMS support their clonal connection in these tumor components.

## Discussion

The tumorigenesis of LMS has long been a topic of debate. While different inciting agents have been implicated in the development of LMS, these etiologies are few and far between [11, 12]. Patients with germline tumor suppressor mutations, notably *RB1* and *TP53* in hereditary retinoblastoma and Li–Fraumeni syndrome, respectively, are at increased risk for the development of LMS [32]. LMS coexisting with benign leiomyoma has been occasionally reported [33]. The theorized nature of myometrial dysplasia, as well as intertumoral areas of LM or LM‐BN, has also been suggested as a harbinger of LMS development [14, 15, 34]. While these studies advanced our knowledge of LMS, only one case series by Mittal *et al* observed intertumoral ‘leiomyoma‐like’ or ‘symplastic leiomyoma‐like’ regions within 26 cases of LMS and demonstrated that these cases sharing genomic alterations of leiomyoma‐like components with LMS [15]. Uterine LM‐BN is a rare variant of leiomyoma [27]. Of the 150 cases reported, LM‐BN shows low recurrence rates and no disease‐related deaths, supporting a benign course [6, 8, 16, 35]. However, LM‐BN is a genomically unstable tumor [4, 8, 36] with increased CNAs, of which many are shared with LMS [30]. *TP53* and *RB1* alterations are common in the cases of LMS with LM‐BN, which has patterns similar to those seen in sporadic LM‐BN and LMS [31, 37, 38]. The molecular relation between LM‐BN and LMS remains unknown.

Herein, we present a case series detailing uterine LMS arising against a background of an associated LM‐BN (Figure 1). These cases had clear‐cut boundaries between the different histologic components, supported by IHC and digital analysis (Figure 2). Of the nine tumors submitted for CNA analysis, all showed regions of shared CNAs in LM‐BN and LMS components, suggesting a similar tumor origin (Figure 3). Increased CNAs were noted in LMS regions, demonstrating high genomic instability toward malignancy. The most shared loci of CNA between the two tumor components were losses in 13q (88.9%) and 17p (66.7%), notably encompassing *RB1* and *TP53*, respectively, while LMS only demonstrated alterations of *CDKN2A/2B*. RNA‐seq spatial transcriptome analysis showed striking geographical expression profile differences between LM‐BN and LMS regions (Figures 4 and 5). These clear‐cut expression differences were further analyzed with cell‐specific cluster analysis, which showcased significant differences in cell expression between LMS and LM‐BN, proving the regional difference of gene expression led to two distinct histologic components. Further assessment of the shared nature of uterine LMS and LM‐BN was achieved by oncogene mutation analysis (Figure 6A). A significant overlap in mutations within each case was identified, further demonstrating the connection between these lesions. The frequently mutated oncogenes in this cohort included *ARID1B*, *BLM*, *PMS2*, *POLE, TP53*, and *RB*, which are major players in numerous pathways associated with tumorigenesis that are involved in DNA repair/recombination and cell cycle regulation. Additionally, missense *NTRK1* mutations were identified in 50% (3/6) of LMS cases; however, these were not within the kinase domain. *NTRK1* rearrangements by gene fusion were previously found in a subset of cervical/uterine sarcomas, but not in LMS [39].

The utilization of AI through cell detection software allowed for the identification of the spatial relationship between LMS, LM‐BN, LM, and myometrium, which could become a supplemental tool for pathologists. These cellular differences and distribution of each tumor component may not always be readily apparent in slide review. Additionally, according to WHO2020, smooth muscle neoplasms with a high density of nuclear atypia and five to nine mitoses/10 HPFs in younger women could be considered to be STUMP, and recent reports detailing IHC workflows have shown the power in using surrogate IHC markers for genetic alterations for challenging cases [40, 41, 42]. Until such next‐generation IHC markers are available, deep learning models may assist with ascertaining selected lesional components that are well established in different cancer studies [43, 44, 45].

Overall, this study provides a comprehensive multimodal histologic and molecular approach to defining the relationship between LMS and LM‐BN in a single mass (Figure 6D) and provides reasonable observations of the histogenesis in a subset of uterine LMS. These findings were also corroborated in our previous study comparing *de novo* LM‐BN and LMS [46]. This study reports the spatially separate regions of LM‐BN and LMS in different tumor development stages characterized by their different transcriptomic and gene mutation patterns, such as *CDKN2A/B* loss in LMS. This is the first study with a comprehensive analysis of the relationship of LMS with adjacent LM‐BN utilizing a multi‐omics approach. Our findings suggest that some LM‐BN can be molecularly related to LMS. The limitation of this study includes its small sample size and further molecular profiling of all cases was not feasible given the nature of cases. While nine of our 11 cases are alive, the relative short‐term follow‐up in these cases precludes a meaningful survival analysis [26].

## Conclusion

In a study of 11 cases, we elucidated the histologic, immunohistochemical, and molecular findings in LMS cases arising from LM‐BN. The molecular analysis identified conserved molecular changes between LMS and LM‐BN, with further progressive genomic alterations identified in LMS components. Transcriptome analysis proved the distinct RNA signatures of the LM‐BN and LMS regions. We argue that a subset of LMS arises from an existing LM‐BN, and highly complex genomic alterations could potentially be a high risk associated with disease progression in LM‐BN. We propose a genomic profiling analysis for all LM‐BN for risk assessment and to potentially improve clinical management.

## Author contributions statement

J‐JW and SEB developed the study concept and design. CF, J‐JW, YF, ZC‐F, MJS and PY developed the methodology. CF, XL, LJJ, MJS and J‐JW acquired, analyzed and interpreted data and performed statistical analyses. J‐JW, CF and YF provided technical and material support. J‐JW, JK and SEB provided funding support. CF and J‐JW contributed to writing, review and revision of the manuscript. All authors read and approved the final submitted version.

## Supporting information


Supplementary materials and methods
**Figure S1.** Representative radiographic and macroscopic gross images for selected cases
**Figure S2.** Histologic and Ki‐67 comparison between LM‐BN and LMS components
**Figure S3.** Representative QuPath digital AI analysis
**Figure S4.** Spatial transcriptome analysis stepwise algorithmic analysis followed a detailed step‐by‐step process to identity spatial expression profiling within LM‐BN and LMS components
**Table S1.** Clinical information for cases
**Table S2A.** Antibody details
**Table S2B.** Immunohistochemical staining patterns in LM‐BN and LMS components
**Table S3.** AI‐based nuclear features in LM‐BN and LMS
**Table S4A.** Chromosomal CNA and LOH in nine cases
**Table S4B.** CNA raw data
**Table S5A.** Summary metrics
**Table S5B.** Top 10 ranking genes and differentially expressed genes between LMS, LM‐BN, and LM detected by spatial transcriptomic analysis
**Table S5C.** Differentially expressed genes in each component
**Table S6A.** Gene mutation types detected using 700 oncogene panel
**Table S6B.** The 700 gene panel

## Data Availability

Spatial transcriptome RNA‐seq data have been deposited in GEO with Accession No. GSE270865 (https://www.ncbi.nlm.nih.gov/geo/query/acc.cgi?acc=GSE270865).

## References

[1] Bell SW , Kempson RL , Hendrickson MR . Problematic uterine smooth muscle neoplasms. A clinicopathologic study of 213 cases. Am J Surg Pathol 1994; 18: 535–558. 8179071

[2] Rubisz P , Ciebiera M , Hirnle L , *et al*. The usefulness of immunohistochemistry in the differential diagnosis of lesions originating from the myometrium. Int J Mol Sci 2019; 20: 1136. 30845657 10.3390/ijms20051136 PMC6429074

[3] Zhang Q , Ubago J , Li L , *et al*. Molecular analyses of 6 different types of uterine smooth muscle tumors: emphasis in atypical leiomyoma. Cancer 2014; 120: 3165–3177. 24986214 10.1002/cncr.28900

[4] Liegl‐Atzwanger B , Heitzer E , Flicker K , *et al*. Exploring chromosomal abnormalities and genetic changes in uterine smooth muscle tumors. Mod Pathol 2016; 29: 1262–1277. 27363490 10.1038/modpathol.2016.107

[5] Guo E , Li C , Hu Y , *et al*. Leiomyoma with bizarre nuclei: a current update. Int J Womens Health 2022; 14: 1641–1656. 36457718 10.2147/IJWH.S388278 PMC9707388

[6] Downes KA , Hart WR . Bizarre leiomyomas of the uterus: a comprehensive pathologic study of 24 cases with long‐term follow‐up. Am J Surg Pathol 1997; 21: 1261–1270. 9351564 10.1097/00000478-199711000-00001

[7] Ly A , Mills AM , McKenney JK , *et al*. Atypical leiomyomas of the uterus: a clinicopathologic study of 51 cases. Am J Surg Pathol 2013; 37: 643–649. 23552381 10.1097/PAS.0b013e3182893f36

[8] Ubago JM , Zhang Q , Kim JJ , *et al*. Two subtypes of atypical leiomyoma: clinical, histologic, and molecular analysis. Am J Surg Pathol 2016; 40: 923–933. 27015034 10.1097/PAS.0000000000000646 PMC5777155

[9] Ip PP , Tse KY , Tam KF . Uterine smooth muscle tumors other than the ordinary leiomyomas and leiomyosarcomas: a review of selected variants with emphasis on recent advances and unusual morphology that may cause concern for malignancy. Adv Anat Pathol 2010; 17: 91–112. 20179432 10.1097/PAP.0b013e3181cfb901

[10] Board WCoTE . Female Genital Tumours (5th edn). WHO, 2020.

[11] Silva EG , Tornos CS , Follen‐Mitchell M . Malignant neoplasms of the uterine corpus in patients treated for breast carcinoma: the effects of tamoxifen. Int J Gynecol Pathol 1999; 13: 248–258. 10.1097/00004347-199407000-00009 7928058

[12] Papatla K , Houck KL , Hernandez E , *et al*. Second primary uterine malignancies after radiation therapy for cervical cancer. Arch Gynecol Obstet 2019; 300: 389–394. 31069490 10.1007/s00404-019-05187-9

[13] George S , Serrano C , Hensley ML , *et al*. Soft tissue and uterine leiomyosarcoma. J Clin Oncol 2018; 36: 144–150. 29220301 10.1200/JCO.2017.75.9845 PMC5759317

[14] Cramer SF , Newcomb PM , Bonfiglio TA . Myometrial dysplasia (atypical myometrial hyperplasia). Hum Pathol 2007; 38: 652–655. 17367607 10.1016/j.humpath.2007.01.010

[15] Mittal KR , Chen F , Wei JJ , *et al*. Molecular and immunohistochemical evidence for the origin of uterine leiomyosarcomas from associated leiomyoma and symplastic leiomyoma‐like areas. Mod Pathol 2009; 22: 1303–1311. 19633649 10.1038/modpathol.2009.96

[16] Croce S , Ducoulombier A , Ribeiro A , *et al*. Genome profiling is an efficient tool to avoid the STUMP classification of uterine smooth muscle lesions: a comprehensive array‐genomic hybridization analysis of 77 tumors. Mod Pathol 2018; 31: 816–828. 29327710 10.1038/modpathol.2017.185

[17] Croce S , Ribeiro A , Brulard C , *et al*. Uterine smooth muscle tumor analysis by comparative genomic hybridization: a useful diagnostic tool in challenging lesions. Mod Pathol 2015; 28: 1001–1010. 25932961 10.1038/modpathol.2015.3

[18] Yang CY , Liau JY , Huang WJ , *et al*. Targeted next‐generation sequencing of cancer genes identified frequent TP53 and ATRX mutations in leiomyosarcoma. Am J Transl Res 2015; 7: 2072–2081. 26692951 PMC4656784

[19] An Y , Wang S , Li S , *et al*. Distinct molecular subtypes of uterine leiomyosarcoma respond differently to chemotherapy treatment. BMC Cancer 2017; 17: 639. 28893210 10.1186/s12885-017-3568-y PMC5594508

[20] Chiang S , Oliva E . Recent developments in uterine mesenchymal neoplasms. Histopathology 2013; 62: 124–137. 23240674 10.1111/his.12048

[21] Oliva E , Zaloudek C , Soslow R . Blaustein's Pathology of the Female Genital Tract (7th edn). Springer Nature Switzerland AG, 2019.

[22] Bertsch E , Qiang W , Zhang Q , *et al*. MED12 and HMGA2 mutations: two independent genetic events in uterine leiomyoma and leiomyosarcoma. Mod Pathol 2014; 27: 1144–1153. 24390224 10.1038/modpathol.2013.243 PMC4081525

[23] Zhang Q , Kanis MJ , Ubago J , *et al*. The selected biomarker analysis in 5 types of uterine smooth muscle tumors. Hum Pathol 2018; 76: 17–27. 29258902 10.1016/j.humpath.2017.12.005 PMC6065267

[24] Bankhead P , Loughrey MB , Fernández JA , *et al*. QuPath: open source software for digital pathology image analysis. Sci Rep 2017; 7: 16878. 29203879 10.1038/s41598-017-17204-5 PMC5715110

[25] Li H , Durbin R . Fast and accurate short read alignment with burrows‐wheeler transform. Bioinformatics 2009; 25: 1754–1760. 19451168 10.1093/bioinformatics/btp324 PMC2705234

[26] Lin JF , Slomovitz BM . Uterine sarcoma 2008. Curr Oncol Rep 2008; 10: 512–518. 18928666 10.1007/s11912-008-0077-9

[27] Croce S , Young RH , Oliva E . Uterine leiomyomas with bizarre nuclei: a clinicopathologic study of 59 cases. Am J Surg Pathol 2014; 38: 1330–1339. 25140893 10.1097/PAS.0000000000000249

[28] Goad J , Rudolph J , Zandigohar M , *et al*. Single‐cell sequencing reveals novel cellular heterogeneity in uterine leiomyomas. Hum Reprod 2022; 37: 2334–2349. 36001050 10.1093/humrep/deac183 PMC9802286

[29] Chudasama P , Mughal SS , Sanders MA , *et al*. Integrative genomic and transcriptomic analysis of leiomyosarcoma. Nat Commun 2018; 9: 144. 29321523 10.1038/s41467-017-02602-0 PMC5762758

[30] Gao T , Finkelman BS , Ban Y , *et al*. Integrated histologic and molecular analysis of uterine leiomyosarcoma and 2 benign variants with nuclear atypia. Cancer Sci 2021; 112: 2046–2059. 33338329 10.1111/cas.14775 PMC8088951

[31] Mehine M , Kaasinen E , Mäkinen N , *et al*. Characterization of uterine leiomyomas by whole‐genome sequencing. N Engl J Med 2013; 369: 43–53. 23738515 10.1056/NEJMoa1302736

[32] Francis JH , Kleinerman RA , Seddon JM , *et al*. Increased risk of secondary uterine leiomyosarcoma in hereditary retinoblastoma. Gynecol Oncol 2012; 124: 254–259. 22027510 10.1016/j.ygyno.2011.10.019 PMC3264733

[33] Lee WY , Tzeng CC , Chou CY . Uterine leiomyosarcomas coexistent with cellular and atypical leiomyomata in a young woman during the treatment with luteinizing hormone‐releasing hormone agonist. Gynecol Oncol 1994; 52: 74–79. 8307505 10.1006/gyno.1994.1014

[34] Mittal K . Precursor lesions for uterine leiomyosarcoma. Hum Pathol 2007; 38: 1289. 10.1016/j.humpath.2007.04.018 17640556

[35] Kefeli M , Caliskan S , Kurtoglu E , *et al*. Leiomyoma with bizarre nuclei: clinical and pathologic features of 30 patients. Int J Gynecol Pathol 2018; 37: 379–387. 28700441 10.1097/PGP.0000000000000425

[36] Anderson ND , Babichev Y , Fuligni F , *et al*. Lineage‐defined leiomyosarcoma subtypes emerge years before diagnosis and determine patient survival. Nat Commun 2021; 12: 4496. 34301934 10.1038/s41467-021-24677-6 PMC8302638

[37] Mills AM , Ly A , Balzer BL , *et al*. Cell cycle regulatory markers in uterine atypical leiomyoma and leiomyosarcoma: immunohistochemical study of 68 cases with clinical follow‐up. Am J Surg Pathol 2013; 37: 634–642. 23552380 10.1097/PAS.0b013e318287779c

[38] Bennett JA , Weigelt B , Chiang S , *et al*. Leiomyoma with bizarre nuclei: a morphological, immunohistochemical and molecular analysis of 31 cases. Mod Pathol 2017; 30: 1476–1488. 28664937 10.1038/modpathol.2017.56 PMC5626591

[39] Chiang S , Cotzia P , Hyman DM , *et al*. NTRK fusions define a novel uterine sarcoma subtype with features of fibrosarcoma. Am J Surg Pathol 2018; 42: 791–798. 29553955 10.1097/PAS.0000000000001055 PMC6764747

[40] Ip PP , Cheung AN , Clement PB . Uterine smooth muscle tumors of uncertain malignant potential (STUMP): a clinicopathologic analysis of 16 cases. Am J Surg Pathol 2009; 33: 992–1005. 19417585 10.1097/PAS.0b013e3181a02d1c

[41] Gupta M , Laury AL , Nucci MR , *et al*. Predictors of adverse outcome in uterine smooth muscle tumours of uncertain malignant potential (STUMP): a clinicopathological analysis of 22 cases with a proposal for the inclusion of additional histological parameters. Histopathology 2018; 73: 284–298. 29537683 10.1111/his.13515

[42] Momeni‐Boroujeni A , Yousefi E , Balakrishnan R , *et al*. Molecular‐based immunohistochemical algorithm for uterine leiomyosarcoma diagnosis. Mod Pathol 2023; 36: 100084. 36788080 10.1016/j.modpat.2022.100084 PMC10191186

[43] Amgad M , Atteya LA , Hussein H , *et al*. NuCLS: a scalable crowdsourcing approach and dataset for nucleus classification and segmentation in breast cancer. Gigascience 2022; 11: giac037. 35579553 10.1093/gigascience/giac037 PMC9112766

[44] Farahani H , Boschman J , Farnell D , *et al*. Deep learning‐based histotype diagnosis of ovarian carcinoma whole‐slide pathology images. Mod Pathol 2022; 35: 1983–1990. 36065012 10.1038/s41379-022-01146-z

[45] Alom Z , Asari VK , Parwani A , *et al*. Microscopic nuclei classification, segmentation, and detection with improved deep convolutional neural networks (DCNN). Diagn Pathol 2022; 17: 38. 35436941 10.1186/s13000-022-01189-5 PMC9017017

[46] Fischer JV , Mejia‐Bautista M , Vadasz B , *et al*. Uterine leiomyosarcoma associated with leiomyoma with bizarre nuclei: histology and genomic analysis of 2 cases. Int J Gynecol Pathol 2022; 41: 552–565. 35093974 10.1097/PGP.0000000000000837 PMC9339039

[47] Mikhail FM , Biegel JA , Cooley LD , *et al*. Technical laboratory standards for interpretation and reporting of acquired copy‐number abnormalities and copy‐neutral loss of heterozygosity in neoplastic disorders: a joint consensus recommendation from the American College of Medical Genetics and Genomics (ACMG) and the cancer genomics consortium (CGC). Genet Med 2019; 21: 1903–1916. 31138931 10.1038/s41436-019-0545-7

[48] Li MM , Datto M , Duncavage EJ , *et al*. Standards and guidelines for the interpretation and reporting of sequence variants in cancer: a joint consensus recommendation of the association for molecular pathology, American Society of Clinical Oncology, and College of American Pathologists. J Mol Diagn 2017; 19: 4–23. 27993330 10.1016/j.jmoldx.2016.10.002 PMC5707196

[49] References [47] and [48] are cited only in supplementary materials

