# Supplementary material for: Genomic characterization and histologic analysis of uterine leiomyosarcoma arising from leiomyoma with bizarre nuclei
Source: J Pathol. 2024 Dec 18;265(2):211–25. doi: 10.1002/path.6379 (PMC11717496; doi:10.1002/path.6379)
Supplement: Supplementary file 1 — Supplementary materials and methods Figure S1. Representative radiographic and macroscopic gross images for selected cases Figure S2. Histologic and Ki‐67 comparison between LM‐BN and LMS components Figure S3. Representative QuPath digital AI analysis Figure S4. Spatial transcriptome analysis stepwise algorithmic analysis followed a detailed step‐by‐step process to identity spatial expression profiling within LM‐BN and LMS components Table S1. Clinical information for cases Table S2A. Antibody details Table S2B. Immunohistochemical staining patterns in LM‐BN and LMS components Table S3. AI‐based nuclear features in LM‐BN and LMS Table S4A. Chromosomal CNA and LOH in nine cases Table S4B. CNA raw data Table S5A. Summary metrics Table S5B. Top 10 ranking genes and differentially expressed genes between LMS, LM‐BN, and LM detected by spatial transcriptomic analysis Table S5C. Differentially expressed genes in each component Table S6A. Gene mutation types detected using 700 oncogene panel Table S6B. The 700 gene panel [file PATH-265-211-s001.zip › path6379-sup-0001-SuppMatMeth,FiguresS1-S4,TablesS1-S6.docx]

**Genomic characterization and histologic analysis of uterine leiomyosarcoma arising from leiomyoma with bizarre nuclei**

C Felicelli *et al. J Pathol* <https://doi.org/10.1002/path.6379>

**Supplementary materials and methods**

**Supplementary Figures S1–S4**

**Supplementary Tables S1–S6**

**Note that Table S4B and Table S5C are provided as separate Excel files**

**Supplementary materials and methods**

Reference numbers refer to the main text list

**Genomic DNA copy number profiling**

Tumor sections from LM-BN and LMS were dissected, and DNA was extracted independently. DNA was subjected to the Affymetrix OncoScan CNA arrays (Thermo Fisher Scientific, Santa Clara, CA, USA). The data were analyzed using Affymetrix Chromosome Analysis Suite (ChAS) software and reviewed by cytogeneticists for copy number alterations (CNAs), including copy number gains or losses, and copy neutral loss of heterozygosity (CN-LOH). The final assessment of somatic CNAs and CN-LOH was performed according to the 2019 guidelines established by the American College of Medical Genetics and Genomics and Cancer Genomics Consortium [47]

**Spatial transcriptome of RNA-sequencing and data analysis**

Cases with the best distributions of both LMS and LM-BN back to back within 6×6 mm regions were selected for spatial transcriptomic RNA-seq. To confirm the RNA quality of each FFPE tissue block, one or two curls (each 10 µm thick) were used for RNA extraction using Qiagen RNeasy FFPE kit (Germantown, MD, USA) following the manufacturer’s protocol. Extracted RNA was examined using an Agilent Bioanalyzer RNA pico-chip to confirm DV200 >30%. Simultaneously, the tissue morphology was examined on H&E-stained slides to identify the presence of both LMS and LM-BN components.

For each FFPE sample, one section (5 µm thick) was placed on Leica Bond plus slides. Each slide was incubated at 42 °C for 3 h followed by overnight room temperature incubation. Slides were stored in desiccated slide holders until proceeding to deparaffinization. The deparaffinization, H&E stains and imaging, and decrosslinking of tissue slides were performed according to 10x Genomics protocol (CG000520 Rev B) specific for Visium CytAssist spatial gene expression for a FFPE kit (Pleasanton, CA, USA). Then the slides were subjected to human probe (v2) hybridization and ligation using 10x Genomics Visium CytAssist spatial gene expression FFPE, human transcriptome, 6.5 mm kit (10x Genomics, PN-1000520). The probes were released from tissue slides and transferred to barcoded Visium slides using the Visium CytAssist instrument followed by probe extension. Sequencing libraries were prepared according to the manufacturer’s protocol. Multiplexed libraries were pooled and sequenced on Novaseq6000 S2 flowcell 100 cycle kit with the following parameters: 28 nt for Read 1 and 90 nt for Read 2. The Visium slide processing, library preparation, and sequencing were done at the Northwestern University NUseq facility core.

Raw sequencing data, in base call format (.bcl), were demultiplexed using Space Ranger (version 2.0.0) from 10x Genomics, with the raw data being converted into FASTQ format. The data were automatically filtered by Cell Ranger (<https://www.10xgenomics.com/support/software/cell-ranger/latest>) to remove barcodes that did not correspond to cells and might have contributed to background noise. Gene exploratory analysis was done based on the SCT-transformed gene expression values, of which a total of 18,051 genes were included. The images were processed and manually aligned using the Loupe Browser (version 5.1.0). Space Ranger was also used for the alignment of the FASTQ files to the human probe set version 2 and to count the number of reads from each cell that aligned with each probe. The resulting matrix files, which summarize the alignment results, were imported to Seurat (Satija Lab, New York Genome Center) for further analysis. In Seurat, each individual sample was preprocessed, normalized, and scaled. All samples were then combined into a single dataset using the Integrate Data function in Seurat, adding metadata to the original sample information. All UMAPs, violin plots, spatial feature expression plots, and heatmaps were generated using Seurat tools. Cell types were automatically assigned using the R package scType.

**Next-Generation Sequencing (NGS)**

NGS was performed to include whole-exon sequencing and a 700-oncogene panel. For exon sequencing, DNA libraries were prepared and performed using a BGISEQ-500 platform (BGI, Cambridge, MA, USA). Genomic DNA was fragmented by Covaris technology to produce a fragment size between 150 and 250 bp. End repair of DNA fragments was performed, and an A base was added at the 3’-end of each strand. Adapters were ligated to both ends of the end-repaired/dA-tailed DNA fragments, then amplified by ligation-mediated PCR (LM-PCR), followed by single-strand separation and cyclization. Rolling circle amplification (RCA) was performed to produce DNA nanoballs (DNBs). Qualified captured libraries were loaded onto the BGISEQ-500 platform, and high-throughput sequencing was performed to ensure that each sample met the average sequencing coverage requirement. Raw data were filtered by removing sequences with the following rules: (1) reads from the adapter, (2) a low-quality base ratio (base quality less than or equal to 5), and (3) an unknown base (N base) ratio of more than 10%. After data cleaning, sequence data from each sample were mapped to the human reference genome (GRCh37/HG19) using Burrows-Wheeler Aligner (BWA) software with a data analysis quality control system (<http://bio-bwa.sourceforge.net>). Variants were initially filtered based on population frequency of alteration with a cutoff of <0.5% population frequency. The variants were filtered to include only exonic and splice site variants. The remaining variants were filtered to exclude synonymous single nucleotide variants. Finally, the pathogenicity of variants was assessed with a combination of ClinVar, Franklin, and OncoKB genomic databases.

Bioinformatics data analysis was performed with Basepair software (<https://www.basepairtech.com/>) using raw sequencing data from the BGISEQ machine. In brief, data were analyzed as follows: reads from cleaned sequence data were mapped to the hg19 (UCSC) and DNA sequences from normal myometrium using BWA (version 0.7.5) with the parameters ‘mem –t 8 -P –M’. Generated files were sorted, and PCR duplicates were removed using Picard (version 1.105) (<http://broadinstitute.github.io/picard>). Subsequently, the BAM files were indexed using samtools (version 0.1.19). The WGS had a quality score of 20 (Q20), and 95–96% of reads were successfully aligned (supplementary material, Figure S2).

Sequencing of the 700-oncogene panel was performed at the Northwestern Diagnostic Molecular Biology Laboratory. Total DNA was extracted from FFPE tumor tissue samples using the manufacturer's instructions (Purigen Biosystems, Pleasanton, CA, USA). DNA quantity was assessed using Qubit™ ds DNA High-Sensitivity Assay kit (Thermo Fisher Scientific, Waltham, MA, USA). Targeted sequencing was performed using Point-n-Seq^TM^ targeted sequencing reagents according to the manufacturer's recommendations (Agilent, Santa Clara, CA, USA). Genomic DNA (100 ng) was sheared mechanically to a target size of 300 bp. Fragmented DNA was end-repaired, phosphorylated, and adenylated. Universal adapters were ligated to the A-tailed DNA molecules, and the adapter-ligated library was purified. The library was hybridized to bridge probes targeting 700 genes. Bridge probes were captured with an anchor probe labeled with biotin- and magnetic streptavidin-coated beads. Sample libraries were purified and indexed using PCR. The library was purified, quantified, and normalized into a sequencing pool and sequenced using a NovaSeq® 6000 and a S1 flowcell (Illumina, San Diego, CA, USA). Sequence data were aligned with BWA1 (version 0.7.17) to human genome version hg38. Variants were called using Vardict2 (version 1.8.3) and Pindel3 (version 0.2.5b). Variants were annotated using wAnnovar4 [25] and filtered as per the Association of Molecular Pathology (AMP) somatic alteration interpretation guidelines [48]. In short, variants were initially filtered based on population frequency of alteration with a cutoff of <0.5% population frequency. Next, the variants were filtered to include only exonic and splice site variants. Then the remaining variants were filtered to exclude synonymous single-nucleotide variants. Finally, the pathogenicity of the variants was assessed with a combination of ClinVar, Franklin, and OncoKB genomic databases.

**Supplementary Figures S1–S4**


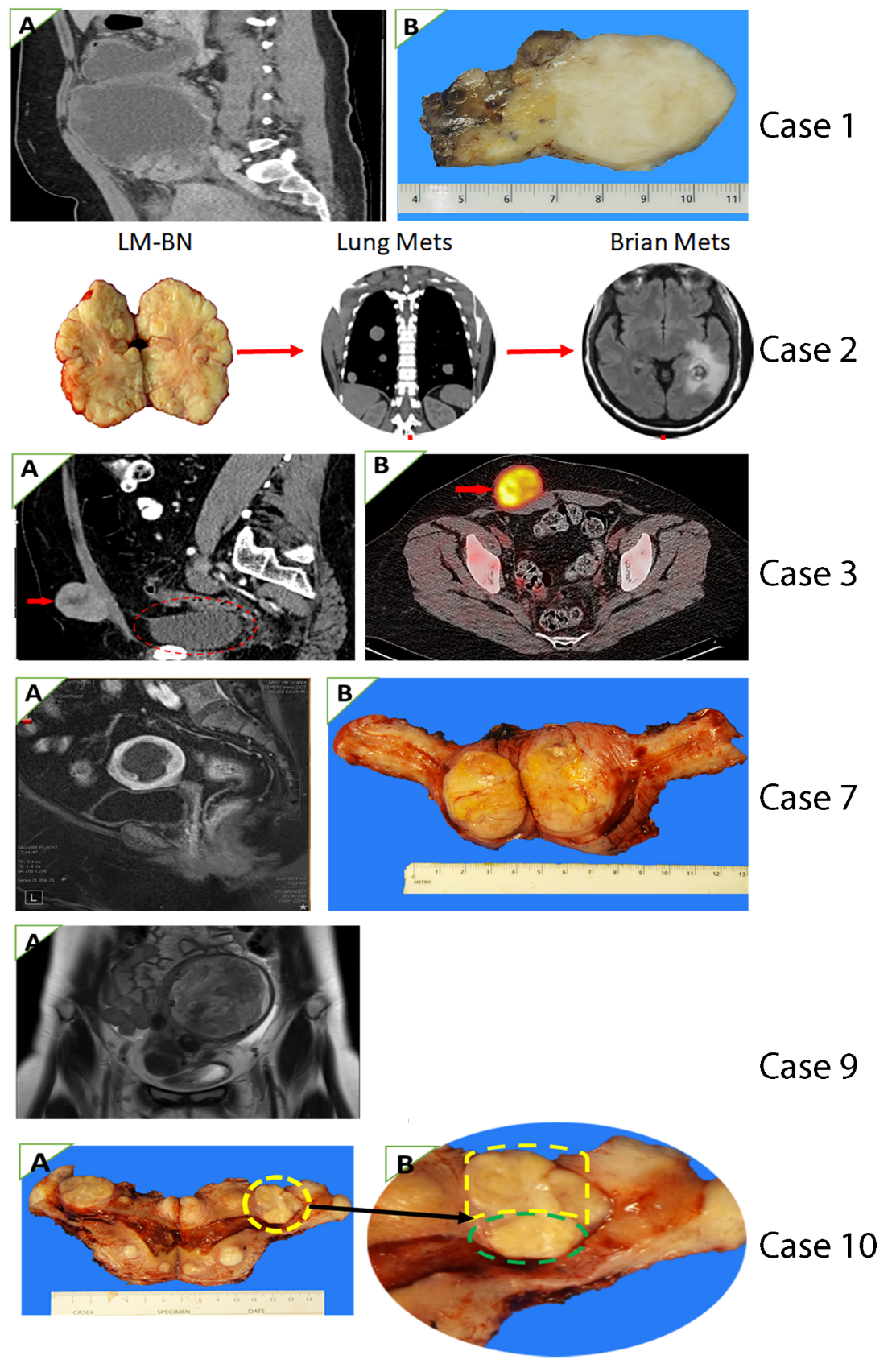


**Figure S1.** Representative radiographic and macroscopic gross images for selected cases.


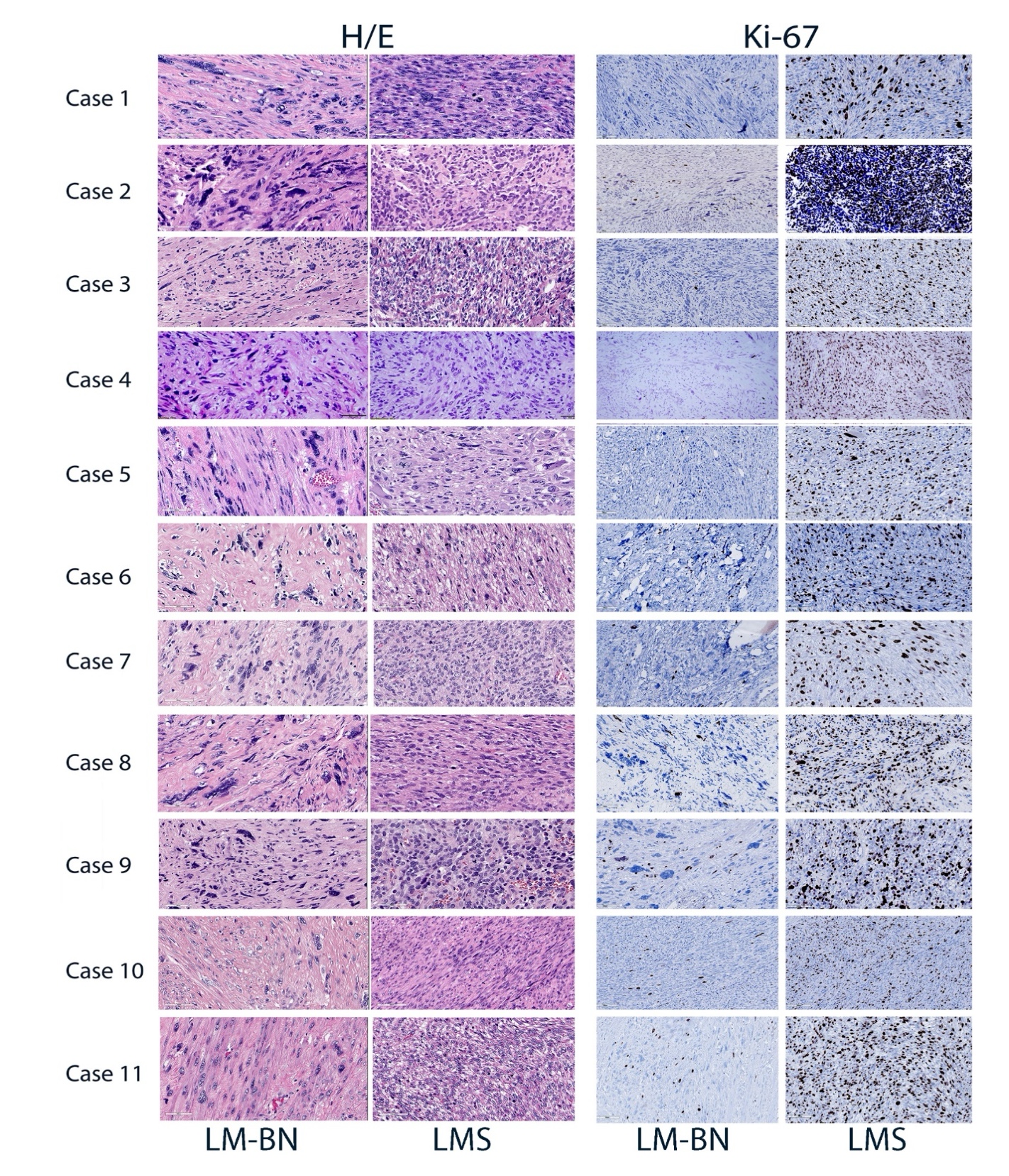


**Figure S2.** Histologic and Ki-67 comparison between leiomyoma with bizarre nuclei (LM-BN) and leiomyosarcoma (LMS) components. In all cases, LM-BN regions were spatially distinct from adjacent LMS regions. LM-BN regions were characterized by striking nuclear atypia, absent necrosis, and minimal mitoses and had a low Ki-67 proliferative index. In comparison, LMS regions showed nuclear atypia, frequent mitoses, and necrosis with a high Ki-67 proliferative index.

**A**

**B**


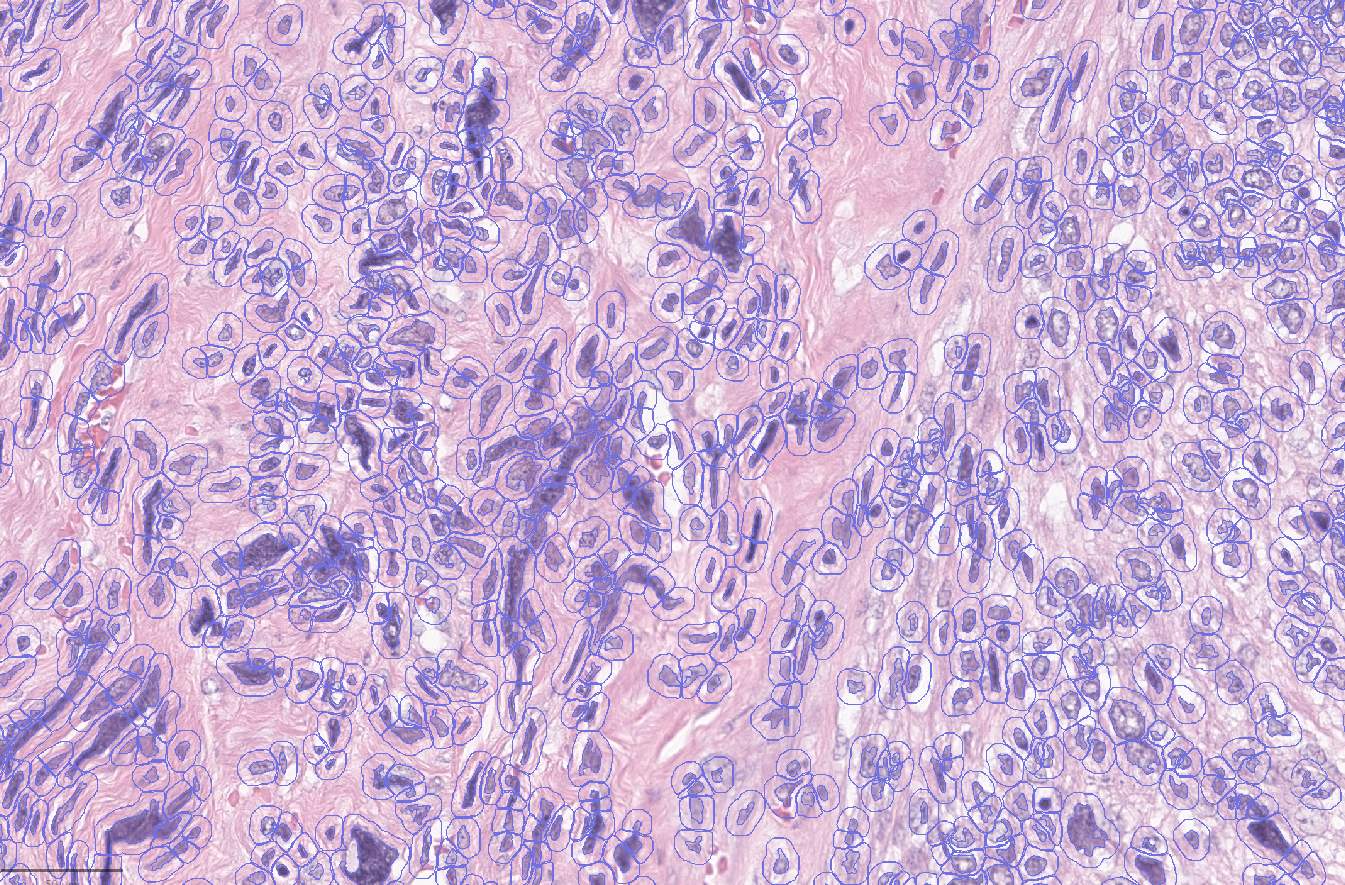

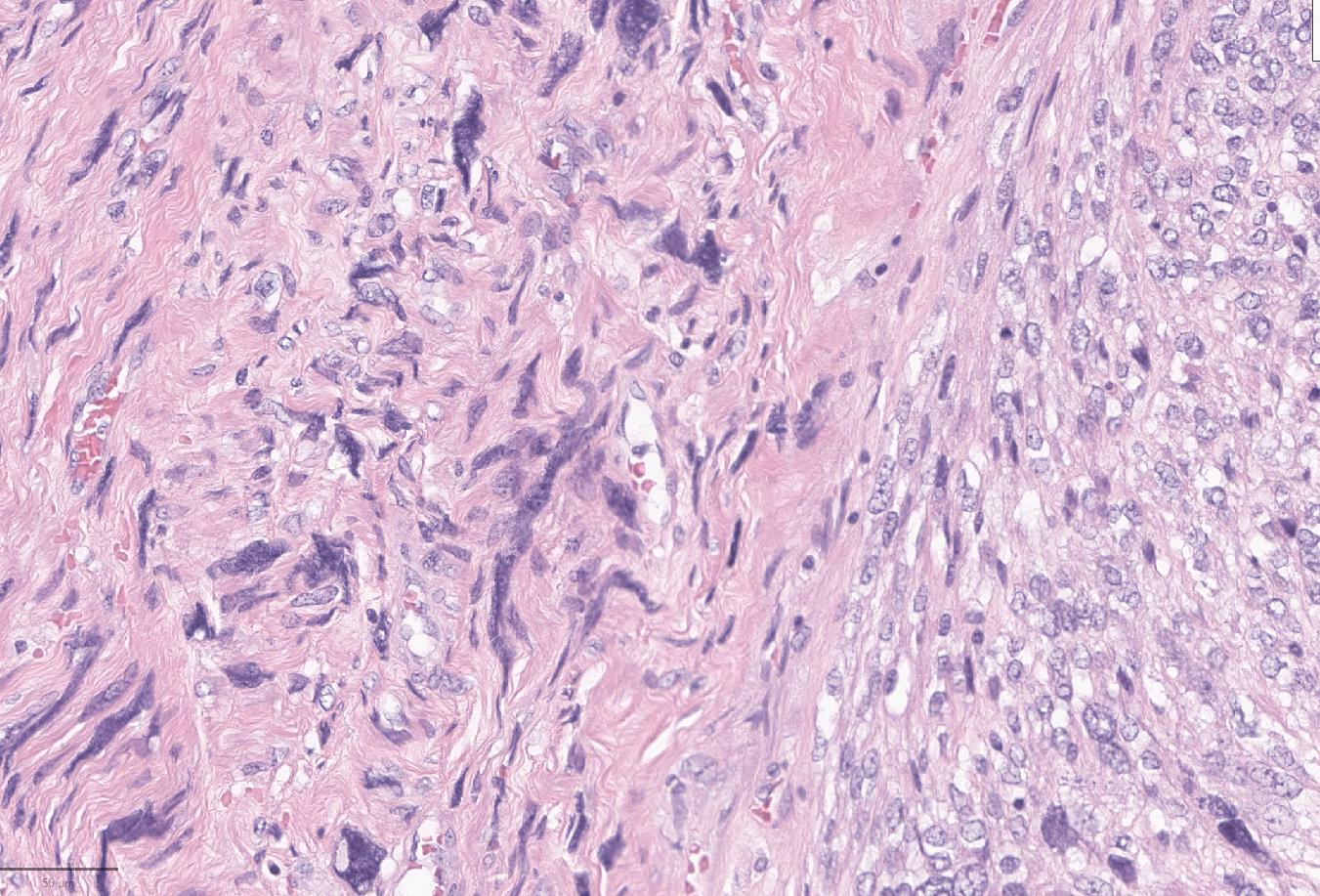

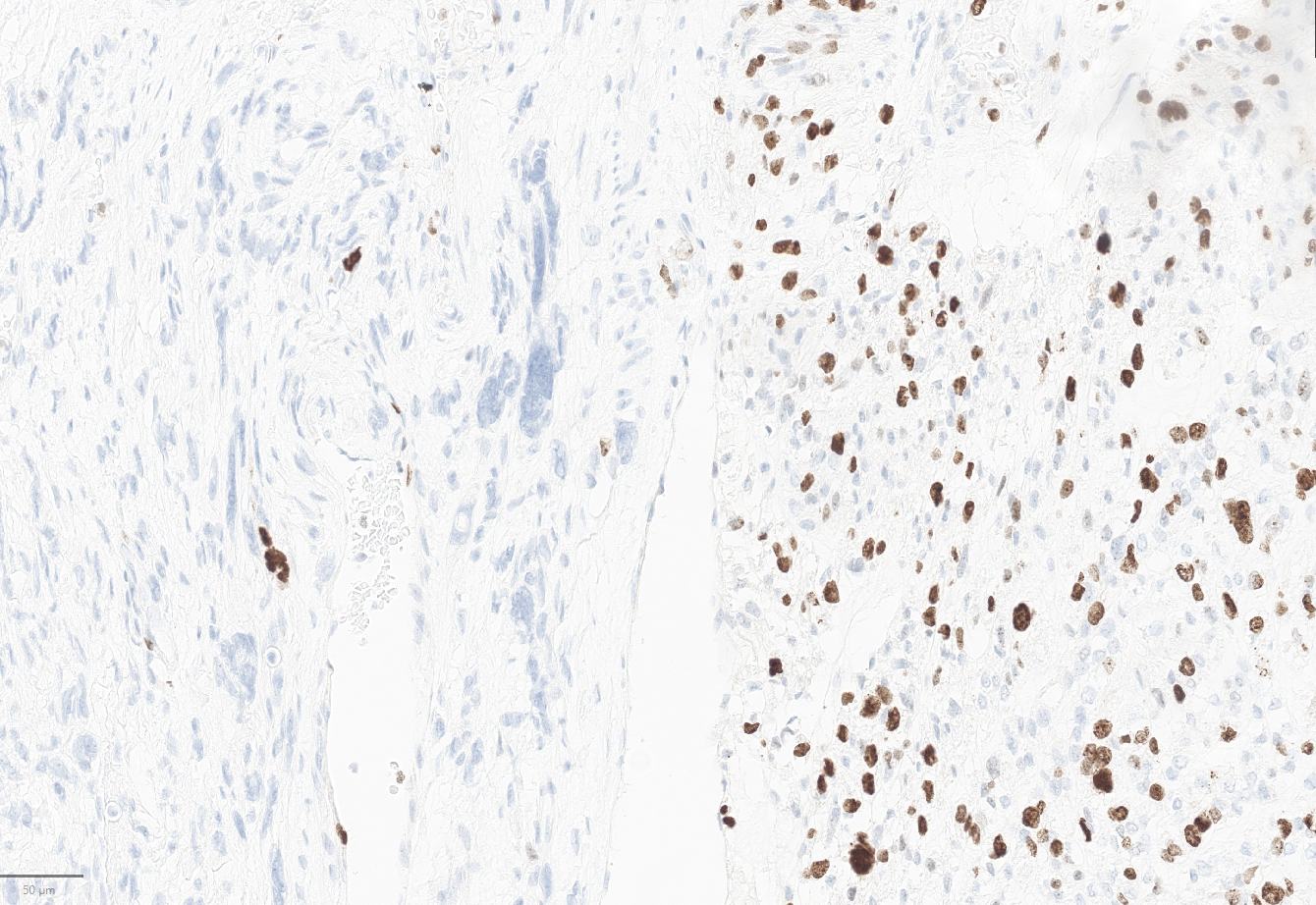

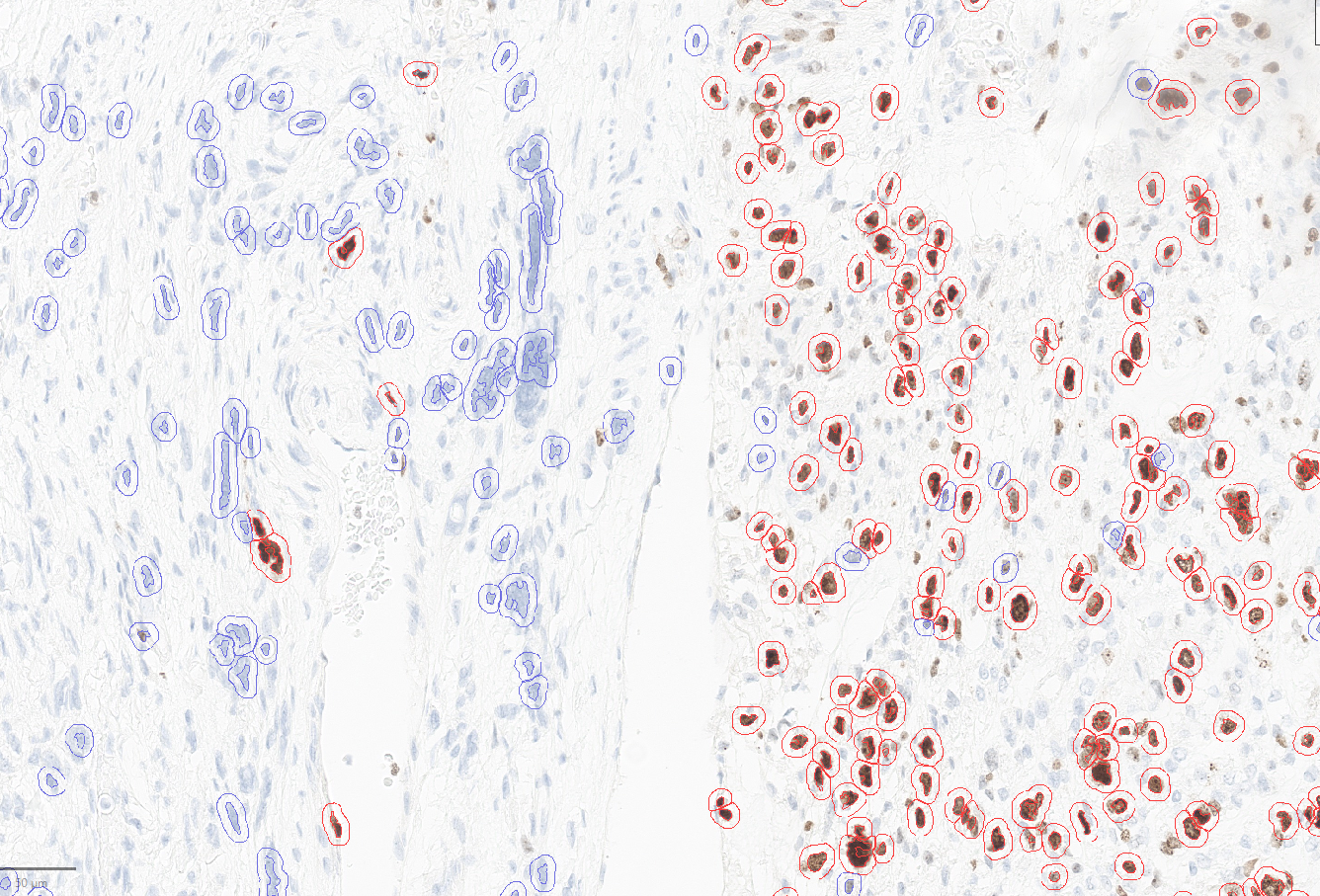


**Figure S3.** Representative QuPath digital AI analysis. (A) QuPath readily detected differences in cellular details, including nuclear and cytoplasmic density. (B) Positive cell detection software can also assess positive for IHC, including Ki-67.


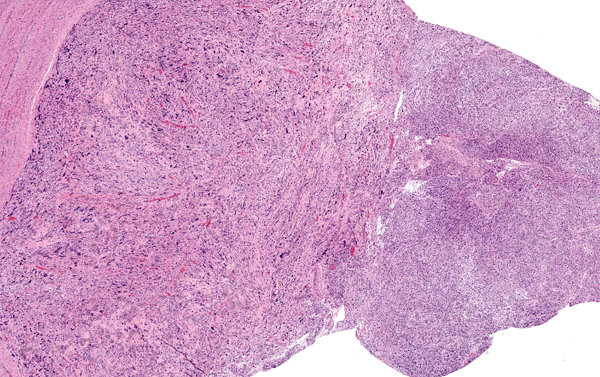


Tissue section


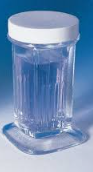


Deparaffinization


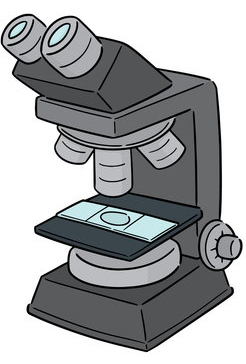


H/E imaging


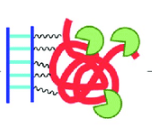


decrosslinking


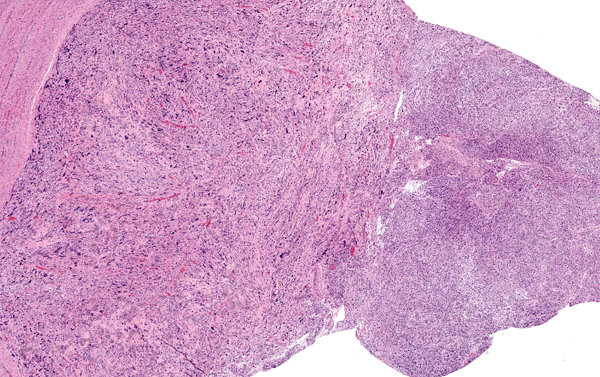

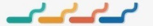

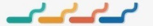


Whole transcriptome Probe-mediated mRNA detection


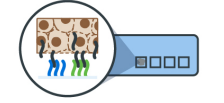


Probe ligation, extension

release and elution


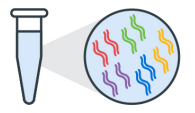


Library construction


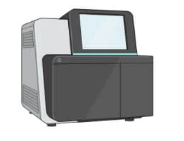


RNA sequencing


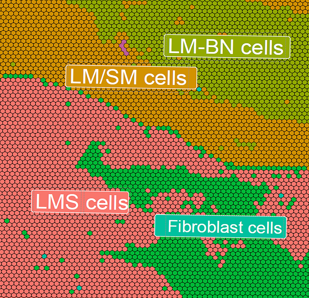


Tissue spatial

Transcriptome map

**Figure S4.** Spatial transcriptome analysis stepwise algorithmic analysis followed a detailed step-by-step process to identity spatial expression profiling within LM-BN and LMS components. Created with BioRender.com.

**Table S1.** Clinical information for cases.

| Case No. | Clinical findings | Gross and histology findings |
| --- | --- | --- |
| 1 | History of abnormal uterine bleeding, seen in emergency department for severe abdominal pain. Abdominal CT revealed large, irregular, and centrally cystic mass in abdomen and pelvis. | Hysterectomy revealed solid and cystic mass attached to left fundus and adherent to omentum. An intraoperative frozen consultation rendered a diagnosis of LMS. Grossly, uterine mass measured 27.0 × 18.5 × 6.4 cm and was composed of heterogeneous, solid, and cystic lesions, consistent with LMS. A 7.5 × 7.0 × 6.2 firm, white-tan, whorled, well-circumscribed mass transitioned into the larger remaining heterogeneous tumor component, consistent with LM-BN. |
| 2 | A past medical history of fibroids. Patient underwent laparoscopic myomectomy in 2012. As part of her follow-up in 2016, a chest X-ray and CT scan demonstrated multiple bilateral pulmonary nodules, with largest in right lower lobe measuring 3.4 cm. Further clinical work-up by MRI demonstrated that she had brain metastases. She was treated with gamma-knife radiosurgery in 2016, 2019, and 2020. She subsequently died of disease. | Myomectomy in 2012 removed nine LMs (1–6 cm), and largest one was diagnosed as LM-BN. The 3.4-cm lung mass was examined by FNA and diagnosed as metastatic LMS. Multiple brain masses were removed and confirmed to be metastatic LMS. |
| 3 | G0P0 with history of uterine LM. She presented with worsening vaginal bleeding and clotting secondary to symptomatic enlarged uterine fibroids. A CT scan showed several additional lesions arising in right lower anterior abdominal wall at site of incision measuring 3.9 × 3.8 × 3.6 cm, a small lesion at right rectus abdominus muscle measuring 1.5 × 1.1 cm, and a mesenteric soft tissue lesion measuring 2.9 × 2.3 × 2.9 cm with central necrotic areas, suspicious for metastatic disease. | The patient underwent total abdominal hysterectomy. Gross examination revealed a uterus with a large bulging tan nodule measuring 7.8 cm. The nodule was serially sectioned to reveal tan-pink whorled cut surfaces with multifocal areas of mild calcification and degenerative change. |
| 4 | G2P2 who presented with abdominal pain. CT scan revealed adnexal mass and heterogeneous soft tissue mass measuring up to 8.9 × 6.5 × 5.2 cm, which was concerning for metastasis. The patient underwent hysterectomy and bilateral salpingo-oophorectomy. | Uterus had one white-tan whorled subserosal LM in posterior wall measuring 1.6 × 1.1 × 0.6 cm. This leiomyoma showed focal pleomorphic and degenerated nuclear atypia. A pelvic/adnexa lesion consisted of a lobulated, 8.1 × 5.1 × 4.7 cm, rubbery mass. |
| 5 | G2P2 with remote history of cervical dysplasia and fibroids who presented with worsening vaginal fullness, dyspareunia, and incomplete emptying of bladder. Pelvic ultrasound revealed large fibroid occupying majority of uterus. Patient underwent hysterectomy with bilateral salpingectomy. | Grossly, a 10.5-cm dominant mass was identified in uterus. |
| 6 | G5P4 with past medical history of fibroids. Patient experienced increased abdominal size for a period of 6 months. She underwent total abdominal hysterectomy and bilateral salpingectomy. | There was a 14.9-cm intramural mass. |
| 7 | G0P0 who presented with postmenopausal bleeding. She underwent pelvic MRI, which revealed 5-cm mass at uterine fundus that was a heterogeneous T2 intermediate signal showing peripheral hyperintense enhancement and central hypointense signals, suggesting necrosis. | Patient underwent exploratory laparotomy. Grossly, uterine mass was well-circumscribed yellow-tan rubbery tumor measuring 4.5 × 4.0 × 3.0 cm with central hemorrhage and necrosis. |
| 8 | G2P1 who presented for abnormal vaginal bleeding. Ultrasound and CT scan revealed multiple submucosal and intramural fibroids with dominant submucosal uterine mass 7 cm in diameter. | Hysterectomy revealed 7.0 × 7.5 × 7.2 cm dominant uterine mass with whorled and multifocal hemorrhage cut surface. |
| 9 | G0P0 who presented for heavy menstrual bleeding and iron deficiency anemia. She had been diagnosed with uterine fibroids 5 years before and had been experiencing bladder and bowel symptoms with pelvic pressure. Her pelvic MRI demonstrated dilated endometrial cavity with abnormal enhancement of right uterine fundus. There were multiple subserosal and intramural nodules ranging from 0.8 to 8.1 cm. Image revealed dominant mass at left fundus within cavity showing altered signal intensity | Patient underwent hysteroscopic myomectomy and bilateral salpingectomy. The endometrium displayed a 3.5-cm white-tan polypoid mass with hemorrhage arising from posterior endomyometrium and fundus. |
| 10 | G3P2012 who presented with painful menstrual cycles. Transvaginal ultrasound revealed three intramural fibroids measuring up to 3.6 cm and normal endometrial stripe measuring 6 mm. The largest fibroid was at junction of lower uterine segment and endocervix. Patient underwent subsequent total laparoscopic hysterectomy and bilateral salpingo-oophorectomy. | Grossly, there were multiple subserosal, submucosal, and intramural tan-white whorled nodules ranging from 0.2 to 3.7 cm. The largest nodule was at junction of lower uterine segment and endocervix with well-demarcated border. Cut surface was uniform tan-white and whorled without necrosis or hemorrhage. It consisted of a mass with two zonal differences, including a large area of tan-yellow and small area of tan-light-brown discoloration. |
| 11 | Presented with urinary retention. Imaging revealed 9-cm intrauterine mass. Staging studies revealed no evidence of distant metastatic disease, and patient was followed with imaging surveillance. Patient then developed lung blebs and subsequent pneumothorax after 18 months of follow-up and underwent lobectomy. Lung biopsy was consistent with metastatic LMS, and she underwent adjuvant chemotherapy. She was referred to our institution 4 years after the initial diagnosis with progressive mediastinal and lung metastasis. | The initial hysterectomy specimen contained a well-circumscribed, 9-cm, tan-yellow mass in posterior lower uterine segment myometrium. Cut surface was rubbery with focal hemorrhage. |

**Table S2A. Antibody details.**

| **Target** | **Vendor** | **Titration** | **Antigen retrieval** |
| --- | --- | --- | --- |
| ER | Roche Ventana, Indianapolis, IN, USA | PD | CC1 |
| PR | Roche Ventana | PD | CC1 |
| P16 | Roche Ventana | PD | CC1 |
| P53 | Roche Ventana | PD | CC1 |
| KI-67 | Roche Ventana | PD | CC1 |
| MELAN A | Roche Ventana | PD | CC1 |
| HMB | Roche Ventana | PD | N/A |
| FH | Santa Cruz Biotechnology, Dallas, TX, USA | 1:200 | ER2 |
| DESMIN | Roche Ventana | PD | Protease 1 |
| ACTIN-SM | Roche Ventana | PD | CC1 |
| H-CALDESMON | Thermo Fisher Scientific, Santa Clara, CA, USA | 1:400 | Epitope Retrieval 2 |

**PD: Prediluted**

**Table S2B. Immunohistochemical staining patterns in LM-BN and LMS components.**

|  | LM-BN | | | | | LMS | | | | |
| --- | --- | --- | --- | --- | --- | --- | --- | --- | --- | --- |
|  | ER  (%) | PR  (%) | p53 | p16 | Ki-67  (%) | ER  (%) | PR  (%) | p53 | p16 | Ki-67  (%) |
| Case 1 | 50% | 50 | Diffuse | Patchy | 5 | 50 | 50 | Diffuse | Patchy | 50 |
| Case 2 | 90% | 90 | Wild type | Diffuse | 10 | 90 | 90 | Wild type | Diffuse | 30 |
| Case 3 | 100% | 100 | Wild type | Diffuse | 5 | 20 | 0 | Diffuse | Diffuse | 30 |
| Case 4 | 90% | - | Diffuse | Diffuse | 5 | 0 | - | Wild type | Diffuse | 50 |
| Case 5 | 50% | 30 | Wild type | Diffuse | 5 | 10 | 0 | Diffuse | Diffuse | 40 |
| Case 6 | 100% | 100 | Null | Diffuse | 5 | 100 | 100 | Null | Diffuse | 50 |
| Case 7 | 80% | 95 | Null | Diffuse | 5 | 50 | 20 | Null | Diffuse | 40 |
| Case 8 | - | - | Diffuse | Patchy | 5 | - | - | Diffuse | Diffuse | 40 |
| Case 9 | 80% | 80 | Diffuse | Diffuse | 5 | 30 | 30 | Diffuse | Diffuse | 50 |
| Case 10 | 80% | 80 | Wild type | Diffuse | 10 | 20 | 20 | Wild type | Diffuse | 60 |
| Case 11 | 80% | 90 | Wild type | Diffuse | 5 | 2 | 2 | Null | Diffuse | 50 |
| Mean | 80% | 79.4 | 54.5%* | 81.8%* | 5.9 | 37.2 | 34.6 | 72.7%* | 90.9%* | 44.5 |

*Percentage of abnormal p53 stain pattern and diffuse p16 positivity.

**Table S3.** AI-based nuclear features in LM-BN and LMS.

|  | LM-BN | | | |  |  | LMS | | | | | |
| --- | --- | --- | --- | --- | --- | --- | --- | --- | --- | --- | --- | --- |
|  | Nuclear Area (mM2) | Nuclear Perimeter (mM) | Nuclear Circularity | Nuclear Eccentricity | Nuclear Chromasia | N:C Ratio | Nuclear Area (mM2) | Nuclear Perimeter (mM) | Nuclear Circularity | Nuclear Eccentricity | Nuclear Chromasia | N:C Ratio |
| 1 | 43.83 | 28.15 | 0.70 | 0.82 | 0.16 | 0.27 | 46.19 | 28.97 | 0.68 | 0.80 | 0.19 | 0.31 |
| 2 | 38.82 | 25.82 | 0.72 | 0.81 | 0.15 | 0.25 | 43.42 | 27.33 | 0.70 | 0.79 | 0.13 | 0.26 |
| 3 | 47.24 | 27.85 | 0.74 | 0.78 | 0.21 | 0.28 | 41.62 | 26.75 | 0.69 | 0.79 | 0.16 | 0.30 |
| 4 | N/A | N/A | N/A | N/A | N/A | N/A | N/A | N/A | N/A | N/A | N/A | N/A |
| 5 | 44.56 | 27.63 | 0.71 | 0.79 | 0.29 | 0.30 | 37.75 | 24.61 | 0.77 | 0.76 | 0.22 | 0.32 |
| 6 | 36.06 | 24.41 | 0.75 | 0.78 | 0.23 | 0.22 | 38.91 | 26.38 | 0.66 | 0.82 | 0.16 | 0.28 |
| 7 | 44.878 | 27.80 | 0.70 | 0.80 | 0.23 | 0.27 | 33.73 | 24.08 | 0.71 | 0.79 | 0.18 | 0.27 |
| 8 | 38.47 | 25.59 | 0.72 | 0.81 | 0.26 | 0.24 | 40.66 | 25.88 | 0.74 | 0.78 | 0.30 | 0.33 |
| 9 | 35.723 | 24.29 | 0.74 | 0.79 | 0.30 | 0.29 | 29.86 | 21.86 | 0.78 | 0.76 | 0.25 | 0.31 |
| 10 | 35.72 | 25.10 | 0.71 | 0.80 | 0.16 | 0.28 | 41.10 | 26.84 | 0.69 | 0.79 | 0.20 | 0.28 |
| Mean | 40.59 ± 0.95 | 26.30 ± 0.35 | 0.72 ± 0.004 | 0.80 ± 0.003 | 0.22 ± 0.01 | 0.26 ± 0.004 | 39.25 ± 0.89 | 25.86 ± 0.37 | 0.71 ± 0.01 | 0.79 ± 0.004 | 0.20 ± 0.01 | 0.30 ± 0.004 |

**Table S4A.** Chromosomal CNA and LOH in nine cases.

| Case | Component | Gain Sites | Loss Sites | LOH | Percentage CNA Shared  (%) | Fraction Genome Alteration  (%) |
| --- | --- | --- | --- | --- | --- | --- |
| 1 | LM-BN  LMS | 0  4 | 4  19 | 14  13 | 41.7 | 5.8  57.0 |
| 2 | LM-BN  LMS | 0  0 | 10  16 | 4  3 | 52.6 | 5.1  22.1 |
| 3 | LM-BN  LMS | 0  3 | 4  15 | 0  0 | 22.2 | 2.3  9.7 |
| 4 | LM-BN  LMS | N/A  N/A | N/A  N/A | N/A  N/A | N/A | N/A  N/A |
| 5 | LM-BN  LMS | 0  14 | 5  19 | 0  5 | 12.8 | 0.9  48.5 |
| 6 | LM-BN  LMS | 0  6 | 3  21 | 0  0 | 11.1 | 1.4  12.8 |
| 7 | LM-BN  LMS | N/A  N/A | N/A  N/A | N/A  N/A | N/A | N/A  N/A |
| 8 | LM-BN  LMS | 1  9 | 9  16 | 0  1 | 49.6 | 10.8  50.1 |
| 9 | LM-BN  LMS | 3  11 | 7  15 | 0  1 | 18.5 | 10.7  49.3 |
| 10 | LM-BN  LMS | 0  1 | 5  12 | 0  0 | 38.5 | 6.0  13.2 |
| 11 | LM-BN  LMS | 8  11 | 15  18 | 3  3 | 76.3 | 9.1  9.4 |
| Total or  (median) | LM-BN  LMS | 12  59 | 62  151 | 21  26 | (29.4) | (5.79  29.11) |

**Table S4B provides CNA raw data as a supplementary Excel file.**

**Table S5A.** Summary metrics

| Case | Spots Under  Tissue | Mean Reads  per Spot | Median Genes  per Spot | Total Reads | Mapping Rate |
| --- | --- | --- | --- | --- | --- |
| 3 | 4,988 | 26,177 | 6,579 | 130,572,517 | 98.80% |
| 5 | 4,904 | 24,430 | 6,410 | 119,802,399 | 98.70% |
| 8 | 4,971 | 38,483 | 5,622 | 191,297,521 | 98.70% |
| 9 | 4,442 | 23,737 | 3,654 | 105,441,003 | 98.90% |

**Table S5B. Top 10 ranking genes and differentially expressed genes between LMS, LM-BN, and LM detected by spatial transcriptomic analysis.**

| **Gene symbol** | **p_val** | **avg_log2FC** | **pct.1** | **pct.2** | **p_val_adj** | **Cluster** |  |
| --- | --- | --- | --- | --- | --- | --- | --- |
| GABRA4 | 0 | 5.79008994 | 0.504 | 0.036 | 0 | Unknown | LMS |
| GPM6B | 0 | 3.46661844 | 0.507 | 0.05 | 0 | Unknown | LMS |
| GABRB1 | 0 | 5.06151381 | 0.509 | 0.069 | 0 | Unknown | LMS |
| MYRF | 0 | 3.43212566 | 0.486 | 0.092 | 0 | Unknown | LMS |
| ARX | 0 | 4.30696951 | 0.346 | 0.021 | 0 | Unknown | LMS |
| PLCH2 | 0 | 3.77181875 | 0.227 | 0.019 | 0 | Unknown | LMS |
| AGMO | 0 | 3.26581537 | 0.219 | 0.025 | 0 | Unknown | LMS |
| C16orf89 | 0 | 3.81899511 | 0.188 | 0.015 | 0 | Unknown | LMS |
| UGT2B11 | 0 | 3.51585197 | 0.168 | 0.015 | 0 | Unknown | LMS |
| OOSP1 | 1.42E-65 | 3.60784657 | 0.029 | 0.002 | 2.57E-61 | Unknown | LMS |
| ELN | 0 | 3.69675561 | 0.829 | 0.228 | 0 | Stromal cells | LM-SM |
| COMP | 0 | 5.32451137 | 0.66 | 0.06 | 0 | Stromal cells | LM-SM |
| INMT | 0 | 4.5304989 | 0.654 | 0.071 | 0 | Stromal cells | LM-SM |
| SPON1 | 0 | 3.70175041 | 0.677 | 0.116 | 0 | Stromal cells | LM-SM |
| LUM | 0 | 3.97484825 | 0.989 | 0.432 | 0 | Stromal cells | LM-SM |
| ITGBL1 | 0 | 5.03852953 | 0.549 | 0.035 | 0 | Stromal cells | LM-SM |
| COL8A1 | 0 | 3.69764313 | 0.562 | 0.094 | 0 | Stromal cells | LM-SM |
| C7 | 0 | 4.2317931 | 0.525 | 0.064 | 0 | Stromal cells | LM-SM |
| CCN2 | 0 | 3.70678875 | 0.998 | 0.608 | 0 | Stromal cells | LM-SM |
| ADH1B | 0 | 5.71567939 | 0.362 | 0.012 | 0 | Stromal cells | LM-SM |
| PLCL12 | 0 | 2.79382224 | 0.732 | 0.266 | 0 | Cancer cells | LM-BN |
| DPP61 | 0 | 2.54447902 | 0.684 | 0.248 | 0 | Cancer cells | LM-BN |
| NEURL1 | 0 | 3.08444494 | 0.509 | 0.124 | 0 | Cancer cells | LM-BN |
| APOD1 | 8.91E-275 | 2.4748896 | 0.541 | 0.171 | 1.61E-270 | Cancer cells | LM-BN |
| IGHG11 | 5.06E-273 | 3.55151577 | 0.436 | 0.106 | 9.13E-269 | Cancer cells | LM-BN |
| IGLC11 | 9.51E-113 | 4.17678027 | 0.133 | 0.022 | 1.72E-108 | Cancer cells | LM-BN |
| IGHG31 | 3.84E-69 | 2.74337395 | 0.143 | 0.037 | 6.92E-65 | Cancer cells | LM-BN |
| PI161 | 3.32E-58 | 2.82109918 | 0.089 | 0.018 | 6.00E-54 | Cancer cells | LM-BN |
| IGLC71 | 2.48E-09 | 2.6473724 | 0.016 | 0.004 | 4.47E-05 | Cancer cells | LM-BN |
| ZNF735 | 1.08E-06 | 2.47464877 | 0.007 | 0.001 | 0.01949759 | Cancer cells | LM-BN |

**Table S5C.** Differentially expressed genes in each component.

This is provided as a separate Excel file.

**Table S6A.** Gene mutation types detected using 700-oncogene panel.

| Gene | Case 1 LM-BN | Case 1 LMS | Case 7 LM-BN | Case 7 LMS | Case 8 LM-BN | Case 8 LMS | Case 11 LM-BN | Case 11 LMS |
| --- | --- | --- | --- | --- | --- | --- | --- | --- |
| RB1 | - | CNV | p.P29del | - | CNV | CNV, p.R656W | CNV, p.N515Kfs | CNV |
| TP53 | p.R26H | CNV | - | - | CNV | CNV, p.P36L | CNV | CNV |
| PMS2 | p.I225M | p.D308Rfs | - | p.I225M | p.R20Q | - | p.T379K | p.R20Q |
| BLM | p.N515Kfs | p.N515Mfs | p.N515Kfs | - | - | p.N515Kfs | p.N515Kfs | - |
| ARID1B | - | - | p.S41del | - | p.S41del | p.S41del | p.S41del | p.S41del |
| CHD2 | - | - | - | - | p.Q1392Tfs | p.Q1392Tfs | p.Q1392Tfs | p.Q1392Tfs |
| SPTA1 | - | - | p.A970D | p.A970D | - | - | - | p.A970D |
| BCL10 | p.I46Yfs | p.I46Yfs | p.I46Yfs | - | - | - | - | p.I46Yfs |
| WRN | p.E510del | - | - | - | - | - | p.E510del | p.E510del |
| POLE | p.V1446Gfs | p.R1286H | p.V1446Gfs | - | - | - | - | - |
| APC | p.N32S | p.R232* | - | - | - | - | - | - |
| ATM | - | - | - | - | p.R13C | p.R2453C | - | - |
| BRCA1 | p.E23Vfs | p.E23Vfs | - | - | - | - | - | - |
| BAX | - | p.E41Gfs | p.E4Gfs | - | - | - | - | - |
| FLCN | p.H429Pfs | p.H429Pfs | - | - | - | p.R179Q | - | - |
| WISP3 | - | p.I298L | - | p.I298L | - | - | - | - |
| CASR | - | - | - | p.A428V | - | - | - | p.A625T |
| NRTK1 |  |  |  | p.G613V |  |  |  | p.G613V |

**Table S6B.** The 700-gene panel.

ABL1, ABL2, ABR, ACVR1, ACVR1B, ACVR2A, ADGRA2, AJUBA, AKAP9, AKT1, AKT2, AKT3, ALK, ALOX12B, ALOX15B, AMER1, ANKRD11, ANKRD26, APC, APLNR, AR, ARAF, ARFRP1, ARHGAP26, ARHGAP35, ARID1A, ARID1B, ARID2, ARID5B, ASXL1, ASXL2, ATM, ATR, ATRX, AURKA, AURKB, AURKC, AXIN1, AXIN2, AXL, B2M, BAP1, BARD1, BAX, BBC3, BCL10, BCL2, BCL2L1, BCL2L11, BCL2L2, BCL6, BCOR, BCORL1, BCR, BEND2, BIRC2, BIRC3, BIRC5, BLM, BMP1, BMPR1A, BRAF, BRCA1, BRCA2, BRD4, BRD7, BRIP1, BTG1, BTG2, BTK, C11ORF30, CALR, CARD11, CASP8, CASR, CBFB, CBL, CBLB, CCND1, CCND2, CCND3, CCNE1, CD22, CD274, CD276, CD38, CD44, CD58, CD70, CD74, CD79A, CD79B, CDC73, CDH1, CDK12, CDK2, CDK4, CDK6, CDK7, CDK8, CDKN1A, CDKN1B, CDKN1C, CDKN2A, CDKN2B, CDKN2C, CEBPA, CENPA, CFTR, CHD2, CHD4, CHD8, CHEK1, CHEK2, CIC, CIITA, CKS1B, COL17A1, CPA1, CRBN, CREBBP, CRKL, CRLF2, CRTC1, CSAD, CSF1R, CSF3R, CSNK1A1, CTC1, CTCF, CTLA4, CTNNA1, CTNNB1, CTRC, CUL3, CUL4A, CUL4B, CUX1, CXCR4, CYLD, CYP17A1, DAXX, DCUN1D1, DDIT3, DDR1, DDR2, DDX3X, DDX41, DDX5, DEFB134, DHX15, DHX9, DICER1, DIS3, DIS3L2, DLX1, DNAJB1, DNMT1, DNMT3A, DNMT3B, DOT1L, DPYD, E2F3, EED, EGFL7, EGFR, EIF1AX, EIF4A2, EIF4E, ELAC2, ELF3, EML4, EP300, EPCAM, EPHA2, EPHA3, EPHA5, EPHA7, EPHB1, EPHB2, EPHB4, ERBB2, ERBB3, ERBB4, ERCC1, ERCC2, ERCC3, ERCC4, ERCC5, ERG, ERRFI1, ESR1, ESR2, ETS1, ETV1, ETV4, ETV5, ETV6, EWSR1, EZH2, EZR, FAM175A, FAM46C, FANCA, FANCC, FANCD2, FANCE, FANCF, FANCG, FANCI, FANCL, FANCM, FAS, FAT1, FBXO11, FBXW7, FGF1, FGF10, FGF12, FGF14, FGF19, FGF2, FGF23, FGF3, FGF4, FGF5, FGF6, FGF7, FGF8, FGF9, FGFR1, FGFR2, FGFR3, FGFR4, FH, FLCN, FLI1, FLT1, FLT3, FLT4, FOXA1, FOXA2, FOXL2, FOXO1, FOXP1, FRS2, FUBP1, FYN, GABRA6, GATA1, GATA2, GATA3, GATA4, GATA6, GEN1, GID4, GLI1, GNA11, GNA13, GNAI2, GNAQ, GNAS, GPC3, GPS2, GRB2, GREM1, GRIN2A, GRM3, GSK3B, H3F3A, H3F3B, H3F3C, HDAC1, HGF, HIF1A, HIST1H1C, HIST1H2BD, HIST1H3A, HIST1H3B, HIST1H3C, HIST1H3D, HIST1H3E, HIST1H3F, HIST1H3G, HIST1H3H, HIST1H3I, HIST1H3J, HIST2H3C, HIST2H3D, HIST3H3, HLA-A, HLA-B, HLA-C, HNF1A, HNRNPK, HOXB13, HOXC6, HRAS, HSD3B1, HSP90AA1, ICOSLG, ID3, IDH1, IDH2, IDO1, IDO2, IFNGR1, IFNGR2, IGF1, IGF1R, IGF2, IKBKE, IKZF1, IKZF3, IL10, IL6R, IL6ST, IL7R, ING1, INHA, INHBA, INPP4A, INPP4B, INSR, IRF1, IRF2, IRF4, IRS1, IRS2, JAK1, JAK2, JAK3, JUN, KAT6A, KDM5A, KDM5C, KDM6A, KDR, KEAP1, KEL, KIAA1549, KIF5B, KIT, KLF2, KLF4, KLHL6, KMT2A, KMT2B, KMT2C, KMT2D, KRAS, LAMP1, LATS1, LATS2, LMO1, LRP1B, LTK, LYN, LZTR1, MAF, MAGEC3, MAGI2, MALT1, MAML1, MAML2, MAP2K1, MAP2K2, MAP2K4, MAP3K1, MAP3K13, MAP3K14, MAP3K4, MAP3K7, MAPK1, MAPK3, MAX, MBD1, MBD4, MC1R, MCL1, MDC1, MDM2, MDM4, MECOM, MED12, MEF2B, MEN1, MERTK, MET, MGA, MGMT, MITF, MKNK1, MLH1, MLH3, MLLT3, MN1, MPL, MRE11A, MSH2, MSH3, MSH6, MST1, MST1R, MTAP, MTOR, MUTYH, MYB, MYBL1, MYC, MYCL, MYCN, MYD88, MYH9, MYOD1, NAB2, NBN, NCOA2, NCOA3, NCOR1, NCOR2, NEGR1, NF1, NF2, NFE2L2, NFKB2, NFKBIA, NKX2-1, NKX3-1, NLRC5, NOTCH1, NOTCH2, NOTCH3, NOTCH4, NPM1, NR3C1, NRAS, NRG1, NSD1, NSD2, NT5C2, NTHL1, NTRK1, NTRK2, NTRK3,NUP93, NUTM1, P2RY8, PAK1, PAK3, PAK7, PALB2, PARG, PARK2, PARP1, PARP2, PARP3, PAX3, PAX5, PAX7, PAX8, PBRM1, PCBP1, PDCD1, PDCD1LG2, PDGFRA, PDGFRB, PDK1, PDPK1, PGR, PHF6, PHOX2B, PIAS3, PIAS4, PIK3C2B, PIK3C2G, PIK3C3, PIK3CA, PIK3CB, PIK3CD, PIK3CG, PIK3R1, PIK3R2, PIK3R3, PIM1, PIM2, PIM3, PLCG1, PLCG2, PLK2, PMAIP1, PML, PMS1, PMS2, PNRC1, POLD1, POLE, POLG, POLQ, POT1, PPARG, PPM1D, PPP2R1A, PPP2R2A, PPP4R2, PPP6C, PRAME, PRC1, PRDM1, PREX2, PRKAR1A, PRKCA, PRKCI, PRKDC, PRSS1, PRSS8, PSIP1, PSMA1, PSMB5, PSMD1, PSMG2, PTCH1, PTEN, PTK2, PTPN11, PTPRD, PTPRO, PTPRS, PTPRT, QKI, QSER1, RAB35, RAC1, RAD21, RAD50, RAD51, RAD51B, RAD51C, RAD51D, RAD52, RAD54L, RAF1, RANBP2, RARA, RASA1, RB1, RBM10, RECQL4, REL, REST, RET, RFWD2, RFX5, RFXAP, RHEB, RHOA, RICTOR, RIT1, RNASEL, RNF43, ROS1, RPL22, RPL5, RPS6KA4, RPS6KB1, RPS6KB2, RPTOR, RRM1, RSPO2, RUNX1, RUNX1T1, RXRA, RYBP, SDC4, SDHA, SDHAF2, SDHB, SDHC, SDHD, SERPINB3, SERPINB4, SETBP1, SETD2, SF3B1, SGK1, SH2B3, SH2D1A, SHQ1, SIN3A, SLC34A2, SLIT2, SLX4, SMAD2, SMAD3, SMAD4, SMARCA1, SMARCA4, SMARCB1, SMARCD1, SMARCE1, SMC1A, SMC3, SMG1, SMO, SNCAIP, SOCS1, SOS1, SOX10, SOX17, SOX2, SOX9, SPEN, SPINK1, SPOP, SPTA1, SRC, SRSF2, STAG1, STAG2, STAT1, STAT3, STAT4, STAT5A, STAT5B, STAT6, STK11, STK40, SUFU, SUZ12, SYK, TAF1, TAF3, TAP1, TAP2, TAPBP, TBL1XR1, TBX3, TCEB1, TCF12, TCF3, TCF7L2, TEK, TERC, TERT, TET1, TET2, TET3, TFE3, TFEB, TFRC, TGFBR1, TGFBR2, TIPARP, TLR4, TMEM127, TMPRSS2, TNFAIP3, TNFRSF14, TOP1, TOP2A, TP53, TP53BP1, TP63, TP73, TRAF2, TRAF3, TRAF7, TRPS1, TSC1, TSC2, TSHR, TYR, TYRO3, U2AF1, UGT1A1, UVRAG, VEGFA, VHL, VTCN1, WHSC1, WHSC1L1, WISP3, WRN, WT1, XBP1, XIAP, XPO1, XRCC2, YAP1, YES1, ZBTB2, ZBTB7A, ZFHX3, ZFP36L1, ZMYM2, ZMYM3, ZNF217, ZNF703, ZNF750, ZRSR2
